# The CUPID (Cultural and Psychosocial Influences on Disability) Study: Methods of Data Collection and Characteristics of Study Sample

**DOI:** 10.1371/journal.pone.0039820

**Published:** 2012-07-06

**Authors:** David Coggon, Georgia Ntani, Keith T. Palmer, Vanda E. Felli, Raul Harari, Lope H. Barrero, Sarah A. Felknor, David Gimeno, Anna Cattrell, Consol Serra, Matteo Bonzini, Eleni Solidaki, Eda Merisalu, Rima R. Habib, Farideh Sadeghian, Masood Kadir, Sudath S. P. Warnakulasuriya, Ko Matsudaira, Busisiwe Nyantumbu, Malcolm R. Sim, Helen Harcombe, Ken Cox, Maria H. Marziale, Leila M. Sarquis, Florencia Harari, Rocio Freire, Natalia Harari, Magda V. Monroy, Leonardo A. Quintana, Marianela Rojas, Eduardo J. Salazar Vega, E. Clare Harris, Sergio Vargas-Prada, J. Miguel Martinez, George Delclos, Fernando G. Benavides, Michele Carugno, Marco M. Ferrario, Angela C. Pesatori, Leda Chatzi, Panos Bitsios, Manolis Kogevinas, Kristel Oha, Tuuli Sirk, Ali Sadeghian, Roshini J. Peiris-John, Nalini Sathiakumar, A. Rajitha Wickremasinghe, Noriko Yoshimura, Danuta Kielkowski, Helen L. Kelsall, Victor C. W. Hoe, Donna M. Urquhart, Sarah Derett, David McBride, Andrew Gray

**Affiliations:** 1 Medical Research Council Lifecourse Epidemiology Unit, University of Southampton, Southampton, UK; 2 School of Nursing, University of São Paulo, São Paulo, Brazil; 3 Corporación para el Desarrollo de la Producción y el Medio Ambiente Laboral – IFA (Institute for the Development of Production and the Work Environment), Quito, Ecuador; 4 School of Engineering, Pontificia Universidad Javeriana, Bogotá, Colombia; 5 Southwest Center for Occupational and Environmental Health, The University of Texas Health Science Center at Houston School of Public Health, Houston, Texas, United States of America; 6 Center for Disease Control and Prevention/National Institute for Occupational Safety and Health, Atlanta, Georgia, United States of America; 7 Medical Research Council Social, Genetic and Developmental Psychiatry Centre, Institute of Psychiatry, Kings College, London, UK; 8 Center for Research in Occupational Health (CiSAL), Pompeu Fabra University, Barcelona, Spain; 9 Carlos III Health Institute: Biomedical Research Networking Center of Epidemiology and Public Health, Granada, Spain; 10 Occupational Health Department, Parc de Salut MAR, Barcelona, Spain; 11 Epidemiology and Preventive Medicine Research Center, University of Insubria, Varese, Italy; 12 Department of Social Medicine, Medical School, University of Crete, Heraklion, Greece; 13 Department of Public health, University of Tartu, Tartu, Estonia; 14 Department of Environmental Health, Faculty of Health Sciences, American University of Beirut, Beirut, Lebanon; 15 Department of Occupational Health, Faculty of Health, Shahroud University of Medical Sciences, Shahroud, Iran; 16 Department of Community Health Sciences, Aga Khan University, Karachi, Pakistan; 17 Department of Medical Education and Health Sciences, Faculty of Medical Sciences, University of Sri Jayewardenepura, Gangodawila, Nugegoda, Sri Lanka; 18 Clinical Research Centre for Occupational Musculoskeletal Disorders, Kanto Rosai Hospital, Kawasaki, Japan; 19 National Institute for Occupational Health, National Health Laboratory Service, Johannesburg, South Africa; 20 Faculty of Health Sciences, University of Witwatersrand, Johannesburg, South Africa; 21 Department of Epidemiology and Preventive Medicine, School of Public Health and Preventive Medicine, Monash University, Melbourne, Victoria, Australia; 22 Department of Preventive and Social Medicine, University of Otago, Dunedin, New Zealand; 23 School of Nursing of Ribeirão Preto, University of São Paulo, São Paulo, Brazil; 24 Federal University of Paraná, Curitiba-PR, Brazil; 25 Institute for Studies on Toxic Substances (IRET), National University of Costa Rica, Heredia, Costa Rica; 26 Department of Occupational and Environmental Health, Università degli Studi di Milano, Milan, Italy; 27 Fondazione Ca’ Granda Ospedale Maggiore Policlinico, Milan, Italy; 28 Department of Psychiatry, Medical School, University of Crete, Heraklion, Greece; 29 Centre for Research in Environmental Epidemiology (CREAL), Barcelona, Spain; 30 IMIM (Hospital del Mar Research Institute), Barcelona, Spain; 31 Consorcio de Investigación Biomédica de Epidemiología y Salud Pública (CIBERESP), Barcelona, Spain; 32 National School of Public Health, Athens, Greece; 33 North Estonia Medical Centre, Tallinn, Estonia; 34 Põlva Hospital, Põlva, Estonia; 35 Klinikum Leverkusen, Leverkusen, Germany; 36 Department of Physiology, Faculty of Medical Sciences, University of Sri Jayewardenepura, Gangodawila, Nugegoda, Sri Lanka; 37 Section of Epidemiology and Biostatistics, School of Population Health, Faculty of Medical and Health Sciences, University of Auckland, Auckland, New Zealand; 38 Department of Epidemiology, School of Public Health, University of Alabama at Birmingham, Birmingham, Alabama, United States of America; 39 Faculty of Medicine, University of Kalaniya, Kelaniya, Sri Lanka; 40 Department of Joint Disease Research, University of Tokyo, Tokyo, Japan; 41 Centre for Occupational and Environmental Health, Department of Social and Preventive Medicine, Faculty of Medicine, University of Malaya, Kuala Lumpur, Malaysia; 42 Injury Prevention Research Unit, Department of Preventive and Social Medicine, University of Otago, Dunedin, New Zealand; Cardiff University, United Kingdom

## Abstract

**Background:**

The CUPID (Cultural and Psychosocial Influences on Disability) study was established to explore the hypothesis that common musculoskeletal disorders (MSDs) and associated disability are importantly influenced by culturally determined health beliefs and expectations. This paper describes the methods of data collection and various characteristics of the study sample.

**Methods/Principal Findings:**

A standardised questionnaire covering musculoskeletal symptoms, disability and potential risk factors, was used to collect information from 47 samples of nurses, office workers, and other (mostly manual) workers in 18 countries from six continents. In addition, local investigators provided data on economic aspects of employment for each occupational group. Participation exceeded 80% in 33 of the 47 occupational groups, and after pre-specified exclusions, analysis was based on 12,426 subjects (92 to 1018 per occupational group). As expected, there was high usage of computer keyboards by office workers, while nurses had the highest prevalence of heavy manual lifting in all but one country. There was substantial heterogeneity between occupational groups in economic and psychosocial aspects of work; three- to five-fold variation in awareness of someone outside work with musculoskeletal pain; and more than ten-fold variation in the prevalence of adverse health beliefs about back and arm pain, and in awareness of terms such as “repetitive strain injury” (RSI).

**Conclusions/Significance:**

The large differences in psychosocial risk factors (including knowledge and beliefs about MSDs) between occupational groups should allow the study hypothesis to be addressed effectively.

## Introduction

Musculoskeletal disorders of the back, neck and upper limb are a major cause of morbidity and disability with substantial economic impact, especially in western countries. In some cases symptoms arise from identifiable pathology in the spine or arm (e.g. a herniated inter-vertebral disc or peripheral nerve compression in the carpal tunnel). Most often, however, the underlying pathology is unclear, and the symptoms are classed as “non-specific”.

Epidemiological research has linked the occurrence of back, neck and upper limb disorders with various physical activities in the workplace [1]–[4], and also with psycho-social risk factors such as low mood and job dissatisfaction [5]–[8]. More recently, evidence has accumulated for a causal role also of “somatising tendency” (i.e. a general tendency to report and worry about common somatic symptoms) [6], [9]. Together, however, these established risk factors do not adequately explain striking temporal changes that have been observed in disability attributed to common musculoskeletal complaints. For example, in Britain rates of incapacity for work because of back problems increased more than sevenfold between 1953 and 1992 at a time when the physical demands of work were generally reducing [10]; and in Australia there was a major epidemic of disability from arm pain during the early 1980s which was not paralleled in other countries where similar technologies and working methods were employed [11].

This gap in understanding has prompted the hypothesis that the development and persistence of non-specific musculoskeletal complaints and resultant disability are importantly influenced by culturally-determined health beliefs as well as by physical activities and mental health [12]. Several observations provide support for a role of health beliefs. For example, among 178 workers carrying out repetitive tasks on an assembly line in Mumbai, India, only one of whom had ever heard of “RSI” (repetitive strain injury), the 12 month prevalence of disabling arm pain (5%) was less than one fifth of that found using the same questions among manual workers in the UK (including those who were of Indian sub-continental origin) [13]. In longitudinal studies of individuals with back and arm pain, negative beliefs about prognosis have proved predictive of their persistence [7], [14]. And in Victoria, Australia, a community-based intervention aimed at modifying people’s beliefs and expectations about back pain was followed by a reduction in morbidity that was not paralleled in a control state [15].

This is not to say that common musculoskeletal symptoms never arise from traumatic injury to tissues. For the most part, however, such injuries would be expected to heal spontaneously over a period of days or weeks, as in other parts of the body. The influence of health beliefs, low mood and somatising tendency is likely to be more on the persistence of symptoms and levels of associated disability than on the occurrence of acute and transient symptoms.

If the hypothesised role of health beliefs were correct, it would have important practical implications. There might be scope for interventions aimed at modifying beliefs and expectations, along the lines of the successful campaign on back pain in Victoria, Australia [15]. More importantly, however, there would be a need for wider review of strategies aimed at preventing work-related musculoskeletal disorders. Currently, preventive efforts focus largely on reduction of physical stresses to the back and arm so as to minimise the risk of injury and maximise opportunities for continued employment in those who have developed symptoms. However, this approach may reinforce beliefs that even quite minor physical stresses (e.g. from use of a computer keyboard) can be seriously hazardous, and might thereby increase workers’ vulnerability to long-term symptoms and disability.

The CUPID (Cultural and Psychosocial Influences on Disability) study was designed to explore further the impact of cultural and psychosocial influences on musculoskeletal symptoms and associated disability. It aims to compare the prevalence of symptoms and disability in workers who are carrying out jobs with similar physical demands, but in a range of cultural environments, and to explore risk factors for the incidence and persistence of symptoms and disability in these varying cultural environments. We here describe the methods by which participants have been recruited and data collected, summarise various characteristics of the study sample, and discuss strengths and limitations of the study method.

## Methods

### Ethical Approval

Ethical approval for the study was provided by the relevant research ethics committee or institutional review board in each participating country (Appendix S1). Written informed consent was obtained from all participants with the following exceptions. For self-administered questionnaires in the UK and Iran, information about the study was provided, and consent to the baseline survey was deemed to be implicit in the return of a completed questionnaire. In Lebanon, according to local practice, oral informed consent was obtained from all participants before interview, and this was recorded on a form signed and dated by the interviewer. In all cases, the method of obtaining consent was approved by the relevant research ethics committee.

### Overview

The study focuses on 47 occupational groups from 18 countries (1–4 groups per country), from which information has been collected by means of an initial baseline questionnaire, followed by a further, shorter questionnaire after an interval of 12 months. Data collection in each country was led by a local investigator, who forwarded anonymised computerised data files to a team at the University of Southampton for collation and analysis (several earlier papers have described analyses based, all or in part, on components of the study in individual countries [16]–[22]). Local investigators also provided background information on the socio-economic circumstances of their study cohorts – for example, on levels of unemployment in the local community and eligibility for sick pay and compensation for occupational injuries.

### Identification and Recruitment of Participants

Local investigators were asked to recruit samples of nurses, office workers who regularly used a computer keyboard and/or mouse, and workers who carried out repetitive manual tasks with their arms or hands. Postal workers sorting mail were identified in advance as a group of manual workers who might be suitable for study, but other sources of manual workers were allowed at the discretion of the local investigator. In one country (Japan), a group of sales and marketing workers was also recruited, and in the presentation and discussion of results, three main categories of occupation are distinguished – nurses, office workers, and “other workers”, the last including the sales and marketing group as well as various manual occupations.

The aim was to restrict the international analysis to workers aged 20–59 years, who had been in their current job for at least 12 months. However, local investigators were free to recruit and carry out local analyses without these restrictions. Initial power calculations indicated that a sample size of 200 workers per occupational group would be more than adequate to detect differences between countries in the prevalence of symptoms and disability of the magnitude that was anticipated, and also for analysis of important risk factors for the incidence and persistence of pain at different anatomical sites in the longitudinal follow-up.

Table 1 describes the occupational groups that were selected for study, and the methods by which participants were identified and the baseline questionnaire administered. In most cases, potentially eligible subjects were identified from employers’ records, sometimes with random sampling to achieve the desired sample size. Some occupational groups provided information at interview, and others by self-completion of questionnaires. In one country (UK), most questionnaires were self-completed, but random sub-samples of each occupational group were instead interviewed.

**Table 1:** Specification and recruitment of study sample.

Country/OccupationalGroup	Detailed description	Method of identification	Method by which baseline questionnaire completed
**SOUTH AND CENTRAL AMERICA**			
**Brazil**			
Nurses	Nurses, nursing technicians and auxiliaries atthe University Hospital in Sao Paolo	Randomly sampled from a listof eligible subjects provided bymanagers	Self-administered (in Brazilian Portuguese)
Office workers	Computer users from an informatics centrein Curitiba	Randomly sampled from a listof eligible subjects provided bymanagers	Self-administered (in Brazilian Portuguese)
Other workers	Sugar cane cutters at a mill in Ribeirao Preto	Randomly sampled from a listof eligible subjects provided bymanagers	Interview (in Brazilian Portuguese)
**Ecuador**			
Nurses	Nursing staff at a Social Security hospital	Quasi-random sampling fromemployment records	Interview (in Spanish)
Office workers	Office workers regular using computers at theMinistry of Public Health in Quito	Quasi-random sampling fromemployment records	Interview (in Spanish)
Other workers	Flower plantation workers in Tabacundo andCayambe, Pichincha	Residents of specified blocks ofbuildings surrounding theflower plantations	Interview (in Spanish)
**Colombia**			
Office workers	Office workers from the Javeriana Universityin Bogota	Quasi-random sampling fromemployment records	Self-administered by web application (In Spanish)
**Costa Rica**			
Nurses	Nurses, auxiliary nurses and nursing assistantsfrom two national hospitals in San Jose	Randomly sampled from payrollrecords	Interview (in Spanish)
Office workers	Office workers from the Central Offices ofthe Costa Rican Social Security System	Randomly sampled from payrollrecords	Interview (in Spanish)
Other workers	Telephone call centre workers at the Duty FreeZone in San Jose	Randomly selected from payrollrecords	Interview (in Spanish)
**Nicaragua**			
Nurses	Nurses in internal medicine, surgery, orthopaedics,gynaecology and paediatrics from two hospitals	Randomly sampled from payrollrecords	Self-administered (in Spanish)
Office workers	Secretaries and accountants with high computeruse at Ministry of Labor and Nicaraguan Instituteof Social Security	Randomly sampled from payrollrecords	Interview (in Spanish)
Other workers	Machine operators from two textilemanufacturing companies	Sample identified from workermembers of the Maria ElenaCuadra Movement	Interview (in Spanish)
**EUROPE**			
**UK**			
Nurses	Nurses from specified wards at SouthamptonUniversity Hospitals NHS Trust	From employment records	Interview for random subsample; remainder by self-administered questionnaire
Office workers	Full-time clerical workers from three departmentsat Houses of Parliament, London	From employment records	Interview for random subsample; remainder by self-administered questionnaire
Other workers	Mail sorters from three Royal Mail centres in theLondon area	From employment records	Interview for random subsample; remainder by self-administered questionnaire
**Spain**			
Nurses	All nurses and nursing assistants employed for at least one year atspecified units of four hospitals inBarcelona.	From employment records	Interview (in Spanish)
Office workers	All office workers from employed for at least oneyear at specified units in four hospitals and oneUniversity (UPF) in Barcelona.	From employment records	Interview (in Spanish)
**Italy**			
Nurses	Nurses and nursing assistants at threehospitals in Milan and Varese	From employment records	Self-administered (in Italian)
Other workers	Production workers at a factory makingpushchairs	From employment records	Self-administered (in Italian)
**Greece**			
Nurses	Nurses at Heraklion University Hospital	Randomly sampled from employment records	Interview (in Greek)
Office workers	Office workers at Heraklion University who were registered as computer users	From employment records	Interview (in Greek)
Other workers	Postal clerks from the central post offices of the four prefectures of Crete	From employment records	Interview (in Greek)
**Estonia**			
Nurses	Nursing staff (nurses, technicians and auxiliaries) at the University Hospital in Tartu and at 31 institutions providing social care	Randomly sampled from lists provided by management	Self-administered (in Estonian or Russian)
Office workers	Secretaries and office workers in specified departments at the University of Tartu	Randomly sampled from lists provided by management	Self-administered (in Estonian or Russian)
**ASIA**			
**Lebanon**			
Nurses	Registered nurses at two hospitals	From employment records	Interview (in Lebanese Arabic)
Office workers	Office workers at an academic institution	From employment records	Interview (in Lebanese Arabic)
Other workers	Production workers at a food manufacturer	From employment records	Interview (in Lebanese Arabic)
**Iran**			
Nurses	Nurses at three university hospitals in Shahroud	Through a nominated manager at each organisation	Self-administered (in Farsi)
Office workers	Office workers at three university hospitals in Shahroud and at four universities in Shahroud (Shahroud University of Medical Sciences, Shahroud University of Technology, Quran Sciences University and Shahroud Azad University)	Through a nominated manager at each organisation	Self-administered (in Farsi)
**Pakistan**			
Nurses	Nurses in in-patient services at Aga Khan University Hospital, Karachi	From employment records	Interview (in Urdu)
Office workers	Full-time hospital receptionists at Aga Khan University Hospital, Karachi	From employment records	Interview (in Urdu)
Other workers	Postal workers from Pakistan Post at two sorting offices in Karachi	Convenience sample of workers from three shifts	Interview (in Urdu)
**Sri Lanka**			
Nurses	Nursing officers at two tertiary care hospitals in Colombo	Randomly sampled from employment records	Interview (in Sinhalese)
Office workers	Computer operators from six companies in Colombo	Randomly sampled from employment records	Interview (in Sinhalese)
Other workers (1)	Postal workers at the Central Mail Exchange in Colombo	Randomly sampled from employment records	Interview (in Sinhalese)
Other workers (2)	Sewing machinists at two garment factories in Colombo District	Randomly sampled from employment records	Interview (in Sinhalese)
**Japan**			
Nurses	Nurses at Tokyo University Hospital	Through a nominated manager	Self-administered (in Japanese)
Office workers	Administrative and clerical workers at Tokyo University Hospital and at four pharmaceutical companies and a private trading company	Through a nominated manager at each organisation	Self-administered (in Japanese)
Other workers (1)	Transportation operatives (mainly lorry drivers and loaders) at two companies transporting baggage and mail	Through a nominated manager at each organisation	Self-administered (in Japanese)
Other workers (2)	Sales/marketing personnel at six pharmaceutical companies	Through a nominated manager at each organisation	Self-administered (in Japanese)
**AFRICA**			
**South Africa**			
Nurses	Nurses at two academic hospitals in Gauteng	From nurses who were at work when wards were visited	Mostly interview with a few self-administered (all in English)
Office workers	Bank workers at a call centre	From lists of workers provided by the employer	Interview (in English)
**AUSTRALASIA**			
**Australia**			
Nurses	Nurses at AlfredHealth (The Alfred, Caulfield Hospital and Sandringham Hospital), Melbourne	From employment records	Self-administered
**New Zealand**			
Nurses	Nurses (Registered, Enrolled or nurse practitioners) on the Nursing Council of New Zealand register	Randomly selected from all nurses holding a current practising certificate	Self-administered
Office workers	People on the 2005 New Zealand electoral roll in jobs likely to involve use of computers in offices	Randomly selected from those on electoral roll with relevant jobs	Self-administered
Other workers	Mail sorters at New Zealand Post	Randomly selected from an employee database	Self-administered

At the time of answering the baseline questionnaire, participants were asked whether they were willing to be re-contacted in the future, and those who agreed were asked (or will be asked) to complete a follow-up questionnaire after an interval of 12 months. In most cases, subjects have been followed up through their place of work, but where this was not possible (e.g. because they had left their original employer), they have been contacted at their home address. In each occupational group, follow-up questionnaires have been completed by the same method (interview or self-administration) as the baseline questionnaire.

### Questionnaires

The baseline questionnaire (Appendix S2) asked about demographic characteristics; education; height; smoking habits; current occupation; pain in different anatomical regions and associated disability for tasks of daily living; awareness of others with musculoskeletal pain; fear-avoidance beliefs concerning upper limb and low back pain; awareness of repetitive strain injury (RSI) or similar terms; distress from common somatic symptoms; mental health; and sickness absence in the past 12 months because of musculoskeletal problems and other types of illness.

The questions about current occupation covered working hours, whether the job involved each of a specified list of physical tasks, and psychosocial aspects of employment such as time pressures and targets, control over work organisation, support, satisfaction and job security. The questions about pain and disability focused on six anatomical regions (low back, neck, shoulder, elbow, wrist/hand and knee) delineated in diagrams, and were similar in wording to questions that had been used successfully in earlier studies, both by self-administration [9], [23], [24] and at interview [13]. The questions on fear-avoidance beliefs were adapted from the Fear Avoidance Beliefs Questionnaire [25]. Questions about distress from somatic symptoms were taken from the Brief Symptom Inventory (BSI) [26], and were chosen to provide a measure of the subject’s tendency to somatise. Questions on mental health were taken from the Short Form-36 (SF-36) questionnaire [27].

The follow-up questionnaire (Appendix S3) asked about: any change of job since baseline and the reasons; recent pain in different anatomical regions and associated disability for tasks of daily living; distress from common somatic symptoms; mental health; and sickness absence in the past 12 months for musculoskeletal and other reasons. Where possible, the wording of questions was identical to that used in the baseline questionnaire.

Both the baseline and follow-up questionnaires were compiled first in English. If necessary, they were then translated into local languages, and the accuracy of the translation was checked by independent back-translation to English. Where this revealed errors, appropriate corrections were made. In addition, in some countries, translated questionnaires were piloted in samples of workers who were not included in the main study, and where this revealed difficulties in understanding, further amendments were made.

Local investigators were at liberty to add to the “core” questions of the international study, and a few (e.g. in Italy, Greece, Iran, Japan, South Africa, Australia and New Zealand) took up this option. However, in doing so, they were asked where possible to place the supplementary questions after the core questions, so as to minimise the chance that they would alter the ways in which participants answered the core questions.

### Group-level Socio-economic Information

As well as individual data on study participants, local investigators also provided standardised information about the socio-economic circumstances of the occupational groups which they had recruited. This included the local unemployment rate at the time of the survey, availability of social security support for the unemployed, entitlement to sick pay in the first three months of absence, entitlement to compensation for work-related musculoskeletal disorders, special financial support for ill-health retirement, fees paid for healthcare, and access to an occupational health service.

## Results

### Response to Baseline Questionnaire

The response to the baseline questionnaire is summarised in Table 2. Participation rates among those invited to take part in the study were greater than 80% in 33 of the 47 occupational groups, ranging from 28% in UK other workers and 39% in Australian nurses to 100% in six occupational groups from Ecuador, Nicaragua, Pakistan and Sri Lanka. However, 2,279 participants were excluded from the international analysis because they fell outside the specified age range (310), had missing data (317), had not worked in their current job for as long as 12 months (783), or (in the case of Australian nurses) were excluded by random sampling (869). After these exclusions, a total of 12,426 workers were available for analysis, with between 92 and 1018 in each occupational group.

**Table 2:** Response to baseline questionnaire.

Country/Occupational Group	Number of subjects approached	Number (%)participated	Number of respondersexcluded	Number of subjects analysed
**Brazil**				
Nurses	200	192 (96%)	7	185
Office workers	300	292 (97%)	11	281
Other workers	300	182 (61%)	89	93
**Ecuador**				
Nurses	252	250 (99%)	31	219
Office workers	250	250 (100%)	7	243
Other workers	282	279 (99%)	52	227
**Colombia**				
Office workers	114	102 (89%)	10	92
**Costa Rica**				
Nurses	275	249 (91%)	29	220
Office workers	275	249 (91%)	26	223
Other workers	252	237 (94%)	32	205
**Nicaragua**				
Nurses	300	300 (100%)	18	282
Office workers	300	300 (100%)	15	285
Other workers	300	300 (100%)	103	197
**UK**				
Nurses	690	290 (42%)	33	257
Office workers	1051	476 (45%)	96	380
Other workers	1569	442 (28%)	56	386
**Spain**				
Nurses	716	687 (96%)	20	667
Office workers	483	471 (98%)	33	438
**Italy**				
Nurses	766	585 (76%)	49	536
Other workers	290	151 (52%)	12	139
**Greece**				
Nurses	240	224 (93%)	0	224
Office workers	202	200 (99%)	1	199
Other workers	154	140 (91%)	0	140
**Estonia**				
Nurses	876	423 (48%)	52	371
Office workers	415	220 (53%)	18	202
**Lebanon**				
Nurses	193	186 (96%)	2	184
Office workers	220	190 (86%)	18	172
Other workers	172	168 (98%)	31	137
**Iran**				
Nurses	263	248 (94%)	2	246
Office workers	213	187 (88%)	5	182
**Pakistan**				
Nurses	250	235 (94%)	48	187
Office workers	216	216 (100%)	36	180
Other workers	235	225 (96%)	3	222
**Sri Lanka**				
Nurses	250	237 (95%)	1	236
Office workers	250	157 (63%)	5	152
Other workers (1)	250	250 (100%)	0	250
Other workers (2)	250	214 (86%)	63	151
**Japan**				
Nurses	1074	814 (76%)	222	592
Office workers	425	346 (81%)	36	310
Other workers (1)	1308	1119 (86%)	101	1018
Other workers (2)	380	372 (98%)	17	355
**South Africa**				
Nurses	280	252 (90%)	5	247
Office workers	285	236 (83%)	7	229
**Australia**				
Nurses	2878	1119 (39%)	869 (excluded because only a random subset of participants was analysed)	250
**New Zealand**				
Nurses	260	181 (70%)	4	177
Office workers	280	146 (52%)	1	145
Other workers	230	116 (50%)	3	113

### Circumstances of Occupational Groups

Table 3 summarises various economic aspects of employment for the occupational groups studied. The local rate of unemployment ranged from <5% in 16 occupational groups to ≥15% in seven. Members of 28 groups would be eligible for social security provision if they became unemployed, although in the three groups from Costa Rica this would be limited to the first three months without a job. Almost all participants could receive some form of sick pay during the first three months of absence from work, but in 22 groups this would not compensate fully for all loss of earnings over that period. Some form of financial compensation for work-related musculoskeletal disorders was available to 40 occupational groups, but 19 groups were ineligible for any special financial support in the event of ill-health retirement.

**Table 3:** Economic aspects of employment.

Country/OccupationalGroup	Local unemploymentrate (%)	Social securityprovision forunemployed	Sick pay in firstthree monthsabsence	Compensation for work-related musculoskeletaldisorders	Special financialsupport for ill-health retirement
**Brazil**					
Nurses	5–9	No	Full for 7 days, butnot up to 3 months	Sometimes	No
Office workers	<5	No	Yes	Usually	Usually
Other workers	≥15	Yes	Partial from outset	Usually	No
**Ecuador**					
Nurses	<5	No	Full for 7 days, butnot up to 3 months	No	No
Office workers	5–9	No	Full for 7 days, butnot up to 3 months	No	No
Other workers	<5	No	Full for 7 days, butnot up to 3 months	No	No
**Colombia**					
Office workers	5–9	No	Yes	Usually	Sometimes
**Costa Rica**					
Nurses	<5	Up to 3 months	Yes	Usually	Usually
Office workers	<5	Up to 3 months	Yes	Usually	Usually
Other workers	<5	Up to 3 months	Yes	Usually	Usually
**Nicaragua**					
Nurses	10–14	No	Yes	Usually	No
Office workers	10–14	No	Yes	Usually	No
Other workers	10–14	No	Yes	Usually	No
**UK**					
Nurses	<5	Yes	Yes	Sometimes	Usually
Office workers	<5	Yes	Yes	Sometimes	Usually
Other workers	5–9	Yes	Yes	Sometimes	Usually
**Spain**					
Nurses	5–9	Yes	Yes	Usually	Sometimes
Office workers	5–9	Yes	Yes	Usually	Sometimes
**Italy**					
Nurses	5–9	Yes	Yes	Sometimes	No
Other workers	5–9	Yes	Yes	Sometimes	No
**Greece**					
Nurses	5–9	Long-term only	Some workers	No	Sometimes
Office workers	5–9	Long-term only	Yes	No	Sometimes
Other workers	5–9	Long-term only	Yes	No	Sometimes
**Estonia**					
Nurses	10–14	Yes	Full from 4 days	Usually	Sometimes
Office workers	10–14	Yes	Full from 4 days	Usually	Sometimes
**Lebanon**					
Nurses	<5	No	Full for 7 days, butnot up to 3 months	Sometimes	Usually
Office workers	5–9	No	Full for 7 days, butnot up to 3 months	Usually	Sometimes
Other workers	5–9	No	Full for 7 days forsome workers, butnot up to 3 months	Sometimes	Sometimes
**Iran**					
Nurses	<5	Most workers	Yes	Sometimes	Sometimes
Office workers	5–9	Most workers	Yes	Sometimes	Sometimes
**Pakistan**					
Nurses	<5	No	Full for 7 days, but not up to 3 months	No	No
Office workers	5–9	No	Full for 7 days, but not up to 3 months	No	No
Other workers	5–9	No	Full for 7 days, but not up to 3 months	No	No

Table 4 describes the access of participants to different sources of healthcare. Most participants had free access to doctors in primary care and hospitals, but fees were more often required for consultation of other health practitioners. All but nine occupational groups were covered by an occupational health service.

**Table 4:** Access to healthcare for musculoskeletal disorders.

Country/Occupational Group	Primary care doctor	Hospital doctor	Other practitioner	Occupational health service	
**Brazil**					
Nurses	Full fee	Full fee	Full fee	Through employer and external	
Office workers	Small fee	Small fee	Small fee	Through employer and external	
Other workers	Free/insured	Free/insured	Free/insured	Through employer	
**Ecuador**					
Nurses	Full fee	Full fee	Full fee	Through employer or external	
Office workers	Full fee	Full fee	Full fee	External	
Other workers	Full fee	Full fee	Full fee	Through employer or external	
**Colombia**					
Office workers	Free/insured	Small fee	Small fee	External	
**Costa Rica**					
Nurses	Free/insured	Free/insured	Free/insured	Through employer and external	
Office workers	Free/insured	Free/insured	Free/insured	Through employer and external	
Other workers	Free/insured	Free/insured	Free/insured	Through employer and external	
**Nicaragua**					
Nurses	Free/insured	Free/insured	Free/insured	External	
Office workers	Free/insured	Free/insured	Free/insured	External	
Other workers	Free/insured	Free/insured	Free/insured	External	
**UK**					
Nurses	Free/insured	Free/insured	Full fee	Through employer	
Office workers	Free/insured	Free/insured	Full fee	Through employer	
Other workers	Free/insured	Free/insured	Full fee	Through employer	
**Spain**					
Nurses	Free/insured	Free/insured	Free/insured	Through employer	
Office workers	Free/insured	Free/insured	Free/insured	Through employer	
**Italy**					
Nurses	Free/insured	Small fee	Full fee	Through employer	
Other workers	Free/insured	Small fee	Full fee	Through employer	
**Greece**					
Nurses	Free/insured	Free/Insured	Varies	No	
Office workers	Free/insured	Free/Insured	Varies	No	
Other workers	Free/insured	Free/insured	Varies	Through employer	
**Estonia**					
Nurses	Free/insured	Small fee	Free/insured	Through employer and external	
Office workers	Free/insured	Small fee	Free/insured	Through employer and external	
**Lebanon**					
Nurses	Full fee	Full fee	Full fee	Through employer	
Office workers	Small fee	Small fee	Small fee	Through employer	
Other workers	Small fee	Small fee	Small fee	Through employer	
**Iran**					
Nurses	Free/insuredor small fee	Free/insuredor small fee	Free/insuredor small fee	Some participants	
Office workers	Free/insuredor small fee	Free/insuredor small fee	Free/insuredor small fee	Some participants	
**Pakistan**					
Nurses	Free/through employer with a cap	Free/through employerwith a cap	Full fee	No	
Office workers	Free/through employer with a cap	Free/through employerwith a cap	Full fee	No	
Other workers	Free/through employer	Free/through employer	Full fee	No	
**Sri Lanka**					
Nurses	Free/insured	Free/insured	Free/insured	No	
Office workers	Free/insured	Free/insured	Free/insured	No	
Other workers (1)	Free/insured	Free/insured	Free/insured	No	
Other workers (2)	Free/insured	Free/insured	Free/insured	No	
**Japan**					
Nurses	Free/insured	Free/insured	Free/insured	Through employer and external	
Office workers	Free/insured	Free/insured	Free/insured	Through employer and external	
Other workers (1)	Free/insured	Free/insured	Free/insured	Through employer and external	
Other workers (2)	Free/insured	Free/insured	Free/insured	Through employer and external	
**South Africa**					
Nurses	Full fee	Small fee	Full fee	Yes	
Office workers	Full fee	Small fee	Full fee	Yes	
**Australia**					
Nurses	Small fee	Small fee	Full fee	Through employer and external	
**New Zealand**					
Nurses	Small fee	Free/insured	Payment varies	External and possibly through employer	
Office workers	Small fee	Free/insured	Payment varies	External and possibly through employer	
Other workers	Small fee	Free/insured	Payment varies	Through employer and external	

### Characteristics of Participants

Table 5 gives information about the demographic characteristics of participants and their hours of work. In all countries, nurses were predominantly female, and in 18 occupational groups more than 90% of subjects were from one sex. Most groups had a broad distribution of ages, but in a few groups, younger (<30 years) or older (≥50 years) workers were less well represented. Levels of education were generally high in nurses and office workers, but lower in many groups of “other workers”. Most subjects had been in their current job for longer than five years, and most worked between 30 and 49 hours per week. However, in Pakistan, Sri Lanka and Japan, the prevalence of longer working hours (>50 hours per week) was high relative to other countries.

**Table 5:** Characteristics of study sample – prevalence (%) by occupational group.

Country/Occupational Group	Sex	Age (years)				Age finished full time education (years)				Years in current job	Hours worked/week		
	Males	20–29	30–39	40–49	50–59	<14	14–16	17–19	20+	>5	<30	30–49	>50
**Brazil**													
Nurses	11.4	15.7	24.9	43.8	15.7	32.6	38.6	13.6	15.2	90.3	5.6	87.2	7.3
Office workers	21.7	1.4	23.1	57.3	18.1	36.9	35.0	17.9	10.2	86.6	50.5	44.7	4.8
Other workers	94.6	32.3	34.4	23.7	9.7	59.1	21.6	12.5	6.8	57.1	0.0	100.0	0.0
**Ecuador**													
Nurses	0.0	6.8	17.8	33.8	41.6	1.8	2.3	29.7	66.2	78.5	73.5	26.5	0.0
Office workers	0.0	11.9	19.8	44.9	23.5	0.4	0.0	35.8	63.8	77.0	3.3	90.5	6.2
Other workers	0.0	43.6	41.4	11.9	3.1	52.0	19.4	11.9	16.7	39.6	2.2	90.3	7.5
**Colombia**													
Office workers	37.0	27.2	44.6	25.0	3.3	0.0	6.5	17.4	76.1	64.1	26.1	64.1	9.8
**Costa Rica**													
Nurses	33.6	32.3	28.2	25.9	13.6	2.3	3.2	22.2	72.2	65.1	0.5	72.1	27.4
Office workers	38.1	32.7	27.8	25.6	13.9	0.5	1.4	21.2	77.0	63.3	1.4	94.6	4.1
Other workers	36.6	49.8	23.4	16.1	10.7	0.0	0.5	27.9	71.6	49.0	16.1	82.4	1.5
**Nicaragua**													
Nurses	3.2	7.4	34.0	37.9	20.6	0.4	2.5	10.7	86.4	88.3	1.1	91.4	7.5
Office workers	27.4	33.3	35.1	22.1	9.5	0.7	4.6	7.4	87.4	57.9	5.3	93.3	1.4
Other workers	54.8	51.8	37.1	7.1	4.1	9.6	24.4	35.0	31.0	21.8	0.0	100.0	0.0
**UK**													
Nurses	10.1	24.5	37.4	26.1	12.1	0.0	23.7	31.9	44.4	73.4	27.6	72.4	0.0
Office workers	44.7	14.7	31.3	32.1	21.8	0.0	11.1	21.6	67.4	62.5	1.6	94.1	4.3
Other workers	62.4	5.4	19.9	36.8	37.8	0.8	31.5	33.3	34.4	85.5	21.8	70.9	7.3
**Spain**													
Nurses	9.9	25.0	29.2	29.4	16.4	0.3	7.8	154	76.5	72.4	11.8	87.3	0.9
Office workers	16.4	16.7	37.7	34.7	11.0	0.0	2.5	21.7	75.8	67.4	11.6	88.1	0.2
**Italy**													
Nurses	16.4	17.5	34.9	32.5	15.1	3.5	11.2	19.4	65.9	79.3	13.1	86.1	0.8
Other workers	28.1	5.0	36.0	37.4	21.6	16.5	33.1	40.3	10.1	83.2	9.6	90.4	0.0
**Greece**													
Nurses	12.1	5.8	67.0	27.2	0.0	0.0	0.4	18.3	81.3	92.0	0.5	97.3	2.3
Office workers	25.1	7.0	46.2	32.7	14.1	0.0	0.0	20.1	79.9	86.4	16.1	71.9	12.1
Other workers	82.9	1.4	12.1	57.9	28.6	2.9	2.1	66.4	28.6	88.6	2.9	92.9	4.3
**Estonia**													
Nurses	0.5	15.1	31.3	26.1	27.5	0.3	10.3	46.7	42.7	70.0	5.8	86.4	7.8
Office workers	15.3	17.3	31.2	27.7	23.8	0.0	0.0	20.5	79.5	66.3	5.0	89.0	6.0
**Lebanon**													
Nurses	33.7	57.6	31.0	9.8	1.6	0.5	0.0	4.9	94.6	48.4	0.0	97.3	2.7
Office workers	42.4	20.3	31.4	30.2	18.0	0.0	1.2	15.1	83.7	70.9	0.0	85.5	14.5
Other workers	52.6	53.3	29.9	12.4	4.4	26.3	29.2	29.9	14.6	47.4	0.0	70.8	29.2
**Iran**													
Nurses	18.3	32.5	46.7	17.9	2.8	0.0	0.8	12.2	87.0	68.7	0.8	65.9	33.3
Office workers	35.2	49.5	34.6	14.8	1.1	0.5	0.5	30.8	68.1	50.0	1.1	63.7	35.2
**Pakistan**													
Nurses	25.7	72.2	23.0	3.7	1.1	0.0	4.3	29.0	66.7	36.4	0.5	26.7	72.7
Office workers	82.2	53.9	34.4	10.6	1.1	0.0	1.7	17.4	80.9	48.0	1.1	35.0	63.9
Other workers	100.0	9.9	22.5	53.6	14.0	0.9	7.8	25.1	66.2	86.9	16.7	77.5	5.9
**Sri Lanka**													
Nurses	0.0	46.2	38.6	12.7	2.5	0.0	0.8	38.6	60.6	50.4	0.0	34.3	65.7
Office workers	71.7	75.7	19.1	2.6	2.6	0.0	0.0	12.5	87.5	30.9	0.0	36.8	63.2
Other workers (1)	100.0	0.4	8.4	46.0	45.2	3.6	65.2	28.0	3.2	81.6	0.0	21.6	78.4
Other workers (2)	0.0	67.5	17.9	10.6	4.0	2.6	29.1	47.0	21.2	40.4	0.0	25.8	74.2
**Japan**													
Nurses	3.4	43.1	32.6	13.5	10.8	0.0	0.0	10.1	89.9	62.5	5.7	59.6	34.7
Office workers	56.5	4.5	36.1	32.9	26.5	0.0	1.3	13.2	85.5	73.9	13.1	50.7	36.3
Other workers (1)	99.6	20.9	40.4	27.4	11.3	0.0	5.7	65.8	28.5	78.3	14.3	15.3	70.5
Other workers (2)	93.2	29.0	50.1	17.7	3.1	0.0	1.4	4.8	93.8	78.3	8.8	12.7	78.5
**South Africa**													
Nurses	3.6	16.2	31.6	37.2	15.0	0.0	0.8	18.0	81.2	69.6	0.0	100.0	0.0
Office workers	32.3	42.8	28.4	20.5	8.3	0.4	11.2	62.3	26.0	41.9	0.0	100.0	0.0
**Australia**													
Nurses	6.8	13.2	29.6	29.2	28.0	0.0	6.8	31.3	61.8	57.8	43.1	48.4	8.5
**New Zealand**													
Nurses	5.6	8.5	21.5	35.6	34.5	0.6	14.7	37.3	47.5	75.7	32.2	62.7	5.1
Office workers	6.2	4.1	12.4	40.0	43.4	0.7	40.7	49.0	9.7	71.7	31.7	64.8	3.5
Other workers	33.6	18.6	17.7	31.0	32.7	0.0	37.2	46.0	16.8	54.9	47.3	51.8	0.9

Table 6 shows the prevalence of different physical tasks by occupational group. As would be expected, a high proportion of office workers (>80% in all but one group) reported using a computer keyboard for longer than four hours per day, while manual lifting of weights ≥25 kg in an average working day was most common in nurses. Patterns of physical activity among the “other workers” were more variable, but several such groups reported a relatively high prevalence of work with the hands above shoulder height.

**Table 6:** Physical activities in an average working day – prevalence (%) by occupational group.

Country/OccupationalGroup	Activity^a^								
	Use keyboard>4 hours	Other repeated wrist/hand movement>4 hours		Repeated elbowbending >1 hour	Hands aboveshoulder height>1 hr	Lifting ≥25 kgby hand		Kneeling/squatting>1 hour	
**Brazil**									
Nurses	9.7	51.9		68.1	11.9	49.7		34.1	
Office workers	70.8	70.8		81.5	12.5	10.3		13.2	
Other workers	0.0	100.0		100.0	0.0	0.0		100.0	
**Ecuador**									
Nurses	8.2	82.6		89	36.1	68.0		62.6	
Office workers	84.0	78.6		84.8	39.1	5.3		16.0	
Other workers	11.5	92.1		95.2	82.4	21.1		79.3	
**Colombia**									
Office workers	90.2	62.0		72.8	18.5	6.5		4.3	
**Costa Rica**									
Nurses	10.9	66.4		82.7	30.9	63.6		44.1	
Office workers	96.0	76.2		84.8	19.3	5.4		9.4	
Other workers	99.0	86.3		88.3	20.5	4.9		4.9	
**Nicaragua**									
Nurses	0.7	78.4		83.0	35.8	42.2		50.0	
Office workers	89.8	91.6		84.9	46.0	13.3		17.2	
Other workers	4.1	73.6		81.7	26.4	13.2		14.7	
**UK**									
Nurses	12.8	44.0		54.9	8.9	28.4		18.7	
Office workers	88.9	31.1		27.1	1.3	4.2		0.5	
Other workers	4.1	81.9		91.2	51.8	12.2		9.8	
**Spain**									
Nurses	18.9	59.4		93.7	52.5	82.2		70.5	
Office workers	96.8	71.0		91.8	27.4	2.1		14.8	
**Italy**									
Nurses	4.9	55.4		80.2	24.6	60.6		17.0	
Other workers	10.1	84.2		85.6	29.5	26.6		4.3	
**Greece**									
Nurses	2.7	71.4		88.8	29.0	70.1		30.4	
Office workers	87.4	58.8		74.9	6.0	7.0		6.5	
Other workers	1.4	83.6		96.4	65.7	47.1		22.1	
**Estonia**									
Nurses	18.1	64.4		72.5	21.0	56.6		28.6	
Office workers	94.6	40.6		51.0	8.4	2.5		2.5	
**Lebanon**									
Nurses	3.3	97.3		96.2	42.9	51.6		34.2	
Office workers	85.5	73.8		77.3	13.4	14.5		7.0	
Other workers	1.5	98.5		97.1	45.3	44.5		25.5	
**Iran**									
Nurses	10.2	63.0		81.3	43.1	24.8		49.6	
Office workers	97.3	89.6		81.3	40.1	7.1		18.7	
**Pakistan**									
Nurses	54.5	93.6		64.2	90.9	73.3		23.0	
Office workers	91.7	95.6		35.6	83.9	24.4		10.0	
Other workers	7.2	78.4		30.2	77.5	25.7		7.2	
**Sri Lanka**									
Nurses	1.3	60.6		43.2	14.4	36.9		9.3	
Office workers	100.0	94.7		72.4	11.8	25.7		17.1	
Other workers (1)	0.0	95.6		95.6	95.6	0.0		0.0	
Other workers (2)	0.7	86.1		60.9	25.2	4.6		29.1	
**Japan**									
Nurses	23.5	23.8		72.8	12.5	66.9		48.5	
Office workers	89.0	12.9		22.6	1.6	3.2		2.3	
Other workers (1)	2.4	32.8		77.8	33.7	83.3		52.3	
Other workers (2)	27.9	10.1		30.1	4.2	9.3		12.1	
**South Africa**									
Nurses	11.3		76.1	85.0	53.4	80.2	26.3		
Office workers	100.0		76.9	78.6	26.2	4.8	1.3		
**Australia**									
Nurses	25.6		32.8	47.6	8.4	25.2	15.2		
**New Zealand**									
Nurses	26.6		32.8	42.4	4.0	31.6	14.1		
Office workers	91.7		40.0	44.8	0.7	2.1	0.0		
Other workers	10.6		87.6	91.2	34.5	51.3	5.3		

Table 7 summarises reported psychosocial aspects of work. Time pressure was common in most occupational groups, but the prevalence of financial incentives to productivity was much more variable. Personal autonomy at work was lowest among “other workers”. Most subjects were satisfied with their jobs, but job dissatisfaction was notably high in Italy, Japan and South Africa. The prevalence of perceived job insecurity ranged from 1.6% in Sri Lankan postal workers to 90.3% in Brazilian sugar cane cutters.

**Table 7:** Psychosocial aspects of work – prevalence (%) by occupational group.

Country/Occupational Group	Incentives^a^	Timepressure^b^	Lack ofchoice^c^	Lack ofsupport^d^	Job dissatisfaction^e^	Perceived jobinsecurity^f^	
**Brazil**							
Nurses	25.4	65.4	13.5	4.9	7.6	20.0	
Office workers	13.9	49.8	9.6	11.7	19.2	24.9	
Other workers	100.0	96.8	96.8	2.2	5.4	90.3	
**Ecuador**							
Nurses	29.2	69.4	39.7	51.6	1.8	30.1	
Office workers	37.0	63.4	10.7	63.4	4.5	29.2	
Other workers	45.8	65.2	52.0	63.4	11.5	50.7	
**Colombia**							
Office workers	50.0	56.5	2.2	40.2	2.2	25.0	
**Costa Rica**							
Nurses	48.2	92.7	24.5	36.8	12.7	17.7	
Office workers	63.2	77.6	8.1	28.7	10.8	18.4	
Other workers	67.8	77.6	50.7	29.3	17.1	26.3	
**Nicaragua**							
Nurses	16.0	72.3	10.3	41.5	13.5	22.7	
Office workers	26.0	80.0	19.3	43.2	9.5	23.2	
Other workers	86.8	60.9	37.1	41.1	6.1	31.0	
**UK**							
Nurses	6.2	75.1	9.7	10.1	14.8	17.9	
Office workers	0.5	76.6	6.8	7.9	7.9	5.0	
Other workers	19.2	79.5	37.8	17.4	15.5	35.8	
**Spain**							
Nurses	21.0	80.1	19.9	77.7	12.0	16.5	
Office workers	26.3	54.3	32.4	78.5	6.6	13.7	
**Italy**							
Nurses	11.6	80.6	13.2	8.2	17.4	21.5	
Other workers	19.4	82.7	53.2	34.5	51.8	41.7	
**Greece**							
Nurses	6.3	97.3	8.9	14.7	33.9	29.0	
Office workers	6.5	83.4	1.5	9.5	7.0	12.6	
Other workers	2.1	97.9	15.0	40.7	18.6	17.9	
**Estonia**							
Nurses	7.8	66.6	23.7	27.0	6.2	14.3	
Office workers	4.0	64.4	2.0	8.4	5.9	23.3	
**Lebanon**							
Nurses	81.0	95.1	6.0	6.5	20.1	38.6	
Office workers	11.6	75.6	7.6	12.2	16.9	25.0	
Other workers	75.9	76.6	29.9	6.6	16.8	41.6	
**Iran**							
Nurses	28.9	90.2	24.8	23.6	29.3	54.9	
Office workers	29.7	74.2	18.7	26.9	26.4	66.5	
**Pakistan**							
Nurses	62.0	96.3	40.1	7.5	9.1	56.7	
Office workers	68.3	96.1	45.6	7.8	7.8	53.9	
Other workers	11.7	95.0	68.0	7.7	9.0	14.9	
**Sri Lanka**							
Nurses	56.8	91.5	5.9	7.2	4.7	11.4	
Office workers	18.4	87.5	10.5	5.3	8.6	43.4	
Other workers (1)	100.0	100.0	0.0	0.0	2.8	1.6	
Other workers (2)	95.4	94.0	17.2	11.9	4.0	33.8	
**Japan**							
Nurses	4.4	63.0	20.9	5.7	44.4	41.2	
Office workers	3.2	35.5	18.1	12.6	70.3	43.5	
Other workers (1)	30.7	81.1	28.0	20.1	41.9	64.5	
Other workers (2)	9.9	41.4	4.5	5.4	69.6	49.6	
**South Africa**							
Nurses	21.1	80.2	23.1	13.8	34.8	29.6	
Office workers	52	95.2	37.6	21.8	43.7	66.4	
**Australia**							
Nurses	4.4	66.8	3.2	7.6	8.8	10.8	
**New Zealand**							
Nurses	1.7	58.2	9.0	8.5	13.6	22.0	
Office workers	2.1	58.6	4.8	18.6	8.3	17.9	
Other workers	34.5	80.5	23.9	14.2	8.8	20.4	

a Either a) piecework or b) payment of a bonus if more than an agreed number of articles/tasks are finished in a day.

b Either a) a target number of articles or tasks to be finished in the day or b) working under pressure to complete tasks by a fixed time.

c Choice seldom or never in all of: a) how work is done, b) what is done at work, and c) work timetable and breaks.

d Support from colleagues or supervisor/manager seldom or never.

e Dissatisfied or very dissatisfied overall.

f Feel job would be rather unsafe or very unsafe if off work for three months with significant illness.

Table 8 shows the proportions of participants who were aware of a term such as “repetitive strain injury” (“RSI”), “work-related upper limb disorder” (“WRULD”) or “cumulative trauma syndrome” (“CTS”), and also the proportions who knew someone else outside work, who had experienced musculoskeletal pain in the past 12 months. Awareness of RSI and similar terms varied widely – from 0.0% in Brazilian sugar cane cutters and 7.0% in South African office workers to 94.6% in Brazilian nurses and 95.9% in New Zealand office workers. There were also marked differences in knowledge of others with musculoskeletal complaints. For example, among food production workers in Lebanon, only 16.1% knew someone outside work with upper limb pain, whereas in telephone call centre workers in Costa Rica, the proportion was 65.9%.

**Table 8:** Awareness of repetitive strain injury (RSI) work related upper limb disorder (WRULD) or cumulative trauma syndrome (CTS) – prevalence (%) by occupational group.

Country/Occupational Group	Proportion (%) of participants reporting awareness of				
	RSI, WRULDor CTS	Someone outside work with pain in past 12 months in			
		Low back	Neck	Upper limb	Knee
**Brazil**					
Nurses	94.6	62.7	49.2	53.0	55.1
Office workers	94.3	60.9	49.1	52.7	50.2
Other workers	0.0	60.2	12.9	36.6	14.0
**Ecuador**					
Nurses	52.1	42.9	34.7	30.1	42.5
Office workers	28.0	50.6	46.1	37.0	42.4
Other workers	24.2	48.0	27.3	39.2	32.2
**Colombia**					
Office workers	43.5	40.2	34.8	32.6	39.1
**Costa Rica**					
Nurses	54.1	55.9	43.6	42.7	46.4
Office workers	26.9	61.0	49.3	48.4	45.7
Other workers	36.1	74.6	65.9	65.9	61.5
**Nicaragua**					
Nurses	56.0	71.6	57.8	58.2	62.8
Office workers	34.0	60.4	54.0	51.2	48.8
Other workers	29.4	41.6	28.4	31.5	26.9
**UK**					
Nurses	76.3	59.1	30.0	35.0	41.2
Office workers	93.7	60	31.8	33.4	42.6
Other workers	47.9	42.5	21.0	26.7	35.0
**Spain**					
Nurses	67.9	82.6	73.1	49.8	55.9
Office workers	59.8	82.9	80.2	45.3	50.6
**Italy**					
Nurses	84.7	82.3	75.6	56.0	55.4
Other workers	77.0	69.8	66.9	54.0	51.1
**Greece**					
Nurses	21.4	82.6	62.5	56.3	50.4
Office workers	24.6	81.4	68.3	64.8	51.3
Other workers	15.7	70.7	50	43.6	36.4
**Estonia**					
Nurses	66.6	69.0	55.3	46.9	57.1
Office workers	49.5	65.8	59.4	47.0	51.5
**Lebanon**					
Nurses	67.9	70.1	58.2	39.1	57.6
Office workers	67.4	56.4	40.7	36.6	32.6
Other workers	34.3	38.7	27.7	16.1	29.2
**Iran**					
Nurses	45.5	76.8	53.3	59.3	69.5
Office workers	25.3	67.0	46.7	54.4	63.2
**Pakistan**					
Nurses	36.9	44.4	23.5	31.0	52.4
Office workers	17.8	39.4	15.0	20	41.1
Other workers	32.4	30.6	19.8	18.9	26.6
**Sri Lanka**					
Nurses	48.3	53.0	40.3	45.8	61.0
Office workers	51.3	45.4	36.8	37.5	47.4
Other workers (1)	82.4	57.2	27.6	36.0	57.2
Other workers (2)	36.4	37.1	20.5	25.2	45.0
**Japan**					
Nurses	72.3	59.5	27.4	35.8	33.6
Office workers	69.4	53.5	28.7	33.5	35.8
Other workers (1)	35.9	51.6	17.5	22.5	20.5
Other workers (2)	70.7	60.8	23.4	27.0	26.8
**South Africa**					
Nurses	47.0	51.4	36.4	34.8	53.8
Office workers	7.0	55.0	38.4	39.3	40.2
**Australia**					
Nurses	78.0	71.6	49.2	49.6	53.2
**New Zealand**					
Nurses	84.7	72.3	53.1	58.2	57.6
Office workers	95.9	64.1	44.8	47.6	54.5
Other workers	86.7	46.9	27.4	37.2	42.5

Table 9 presents the prevalence of potentially adverse health beliefs about back and arm pain by occupational group. These again varied substantially (more than tenfold) between occupational groups. For example, 78.6% of Greek postal workers and 77.7% of Lebanese nurses believed that low back pain is commonly caused by people’s work, as compared with only 4.0% of Sri Lankan postal workers and no Brazilian sugar cane cutters; and 31.4% of Brazilian nurses and 31.0% of Brazilian office workers had pessimistic views about the prognosis of arm pain, as compared with 1.6% of nurses and office workers in Iran and 0.0% of Brazilian sugar cane cutters.

**Table 9:** Adverse health beliefs regarding low back and arm pain – prevalence (%) by occupational group.

	Low back pain			Arm pain			
Country/Occupational Group	Commonly causedby people’s work^a^	Physical activityis harmful^b^	Poor prognosis^c^	Commonly causedby people’s work^a^	Physical activity is harmful^b^	Poor prognosis^c^	
**Brazil**							
Nurses	25.9	5.9	29.7	31.9	7.0	31.4	
Office workers	32.7	7.5	31.3	42.7	6.0	31.0	
Other workers	0.0	1.1	0.0	0.0	1.1	0.0	
**Ecuador**							
Nurses	53.9	25.1	20.5	52.1	18.7	20.5	
Office workers	37.9	18.9	10.7	33.7	16.0	9.9	
Other workers	77.1	36.1	4.0	76.2	27.3	5.3	
**Colombia**							
Office workers	12.0	1.1	13.0	13.0	1.1	13.0	
**Costa Rica**							
Nurses	30.0	10.9	17.7	35.0	10.5	19.1	
Office workers	13.9	4.0	24.2	11.7	2.7	22.0	
Other workers	16.1	2.9	25.9	18.0	2.0	21.5	
**Nicaragua**							
Nurses	36.2	23.8	15.2	35.5	21.3	14.5	
Office workers	29.1	11.9	9.5	32.3	12.6	9.1	
Other workers	38.1	22.3	10.7	36.5	16.8	8.6	
**UK**							
Nurses	23.7	9.3	5.8	15.2	3.5	2.7	
Office workers	9.2	2.9	4.7	10.8	1.3	3.2	
Other workers	25.6	10.4	8.8	20.7	5.2	5.7	
**Spain**							
Nurses	46.8	23.8	28.2	36.1	13.8	18.3	
Office workers	22.4	15.5	22.1	19.6	9.6	15.3	
**Italy**							
Nurses	34.1	3.2	6.9	24.1	0.9	4.5	
Other workers	36.0	7.9	15.8	40.3	3.6	16.5	
**Greece**							
Nurses	73.2	49.1	14.7	68.3	33.5	12.9	
Office workers	40.2	31.2	10.6	44.2	18.6	12.6	
Other workers	78.6	68.6	20.0	76.4	47.1	12.9	
**Estonia**							
Nurses	27.5	9.2	7.5	25.9	5.9	5.9	
Office workers	15.8	2.5	11.4	21.3	0.5	10.9	
**Lebanon**							
Nurses	77.7	43.5	27.2	62.5	23.9	9.8	
Office workers	36.6	24.4	15.1	36.0	11.0	7.6	
Other workers	66.4	77.4	14.6	59.9	57.7	6.6	
**Iran**							
Nurses	31.7	11	2.8	24.8	4.1	1.6	
Office workers	24.2	12.1	4.9	22.0	2.7	1.6	
**Pakistan**							
Nurses	51.9	50.3	5.9	47.1	26.2	4.8	
Office workers	54.4	43.3	3.9	38.9	29.4	1.7	
Other workers	40.5	31.5	5.9	36.9	28.4	6.3	
**Sri Lanka**							
Nurses	5.9	6.4	9.3	9.7	3.0	11.4	
Office workers	13.8	10.5	4.6	19.7	4.6	3.9	
Other workers (1)	4.0	36.0	10.4	3.6	11.2	8.0	
Other workers (2)	20.5	9.9	7.3	20.5	6.0	6.0	
**Japan**							
Nurses	46.6	14.7	18.2	24.3	5.7	9.3	
Office workers	16.5	19.7	14.2	11.6	9.0	7.4	
Other workers (1)	47.2	25.6	21.8	33.2	11.7	10.1	
Other workers (2)	21.4	23.7	17.5	12.4	16.1	6.5	
**South Africa**							
Nurses	37.7	5.3	7.7	36.0	3.6	6.1	
Office workers	24.9	6.6	4.8	22.7	3.1	3.5	
**Australia**							
Nurses	19.2	2.8	6.8	12.4	2.4	2.4	
**New Zealand**							
Nurses	20.3	2.8	2.3	11.9	1.1	4.0	
Office workers	6.2	2.1	2.8	9.0	2.1	4.1	
Other workers	21.2	14.2	6.2	29.2	12.4	5.3	

a Completely agree that such pain is commonly caused by people’s work.

b Completely agree that for someone with such pain, a) physical activity should be avoided as it might cause harm, and b) rest is needed to get better.

c Completely agree that for someone with such pain, rest is needed to get better, and completely disagree that such problems usually get better within three months.

Table 10 compares the characteristics of participants in the UK who answered the questionnaire at interview and by self-administration. Among the nurses and especially the “other workers”, participation rates were higher among those invited to interview, whereas in the office workers they were slightly lower. However, there were no consistent differences in the prevalence of reported occupational activities and musculoskeletal pain according to the method of data collection.

**Table 10:** Comparison of UK participants who provided information by interview and by self-administered questionnaire.

	Nurses		Office workers		Other workers	
	Interview	Self-administered questionnaire	Interview	Self-administered questionnaire	Interview	Self-administered questionnaire
**Number selected**	190	500	200	851	240	1329
**Number (%) participated**	91 (48)	199 (40)	88 (44)	388 (46)	122 (51)	320 (24)
**Number of subjects analysed**	78	179	66	314	110	276
**Prevalence (%) of activities in an average working day**						
Use keyboard >4 hr	6.4	15.6	84.9	89.8	1.8	5.1
Other repeated wrist/hand movement >4 hr	46.2	43.0	22.7	32.8	86.4	80.1
Repeated elbow bending >1 hr	60.3	52.5	13.6	29.9	96.4	89.1
Hands above shoulder height >1 hr	7.7	9.5	1.5	1.3	55.5	50.4
Lifting ≥25 kg by hand	28.2	28.5	9.1	3.2	12.7	12.0
Kneeling/squatting >1 hr	21.8	17.3	1.5	0.3	15.5	7.6
**Prevalence (%) of pain in past month**						
Low back	26.9	36.3	28.8	26.8	34.6	34.4
Neck	14.1	20.1	21.2	22.9	20.9	20.7
Shoulder	9.0	21.8	21.2	20.7	33.6	31.2
Elbow	2.6	2.8	12.1	8.0	14.6	15.2
Wrist/hand	14.1	15.6	19.7	17.5	24.6	21.7
Knee	12.8	18.4	27.3	22.3	21.8	24.6

## Discussion

The CUPID study has generated substantial information which will be the subject of multiple reports. A particular strength is its use of standardised questions to collect information from participants in many different countries and cultural settings. This should provide valuable insights into the determinants of common musculoskeletal illness and associated disability, and particularly the extent of differences between countries.

The occupational groups were chosen for study with the aim that the prevalence of relevant physical tasks should differ between the three broad categories (nurses, office workers and “other workers”), but that within each of these categories, it should be broadly similar across countries. For nurses and office workers this objective was fairly well achieved, although inevitably there was some heterogeneity. For example, in some countries, nurses routinely lift and move patients, whereas in others such tasks may normally be undertaken by care assistants or patients’ family members. For “other workers”, there was more variation in occupational activities, reflecting the greater diversity of groups selected for study. Nevertheless, the mix of activities tended to differ from that of nurses and office workers, with a relatively high prevalence of work with the arms elevated; and apart from sales personnel in Japan, all groups of “other workers” had a high prevalence of work involving prolonged repetitive movement of the wrists or hands.

The international analysis of data is restricted to subjects aged 20–59 years at baseline, who had held their current job for at least 12 months. These restrictions were set when the CUPID study was first planned, the latter because some outcomes of interest from the baseline survey, such as sickness absence in the past 12 months, would otherwise be difficult to interpret.

The questions used in the baseline and follow-up surveys were for the most part well-established, having been used successfully in previous studies. In particular, the items on mental health and somatising tendency were taken from validated instruments, and have previously demonstrated predictive validity for the incidence and persistence of musculoskeletal symptoms [7]. Similarly, the questions on fear avoidance beliefs were based on a validated questionnaire [25], and have shown predictive validity in a longitudinal study [7]. The questions on occupational physical activities have been successfully used in earlier studies [7], [13], [23], [24], and the consistency of answers with expectation (e.g. the high prevalence of prolonged keyboard use in office workers) supports their validity. There is no reliable standard against which to assess the accuracy with which subjective symptoms such as pain are reported, but the questions about pain and disability had again been used successfully in earlier studies. Moreover, the style of our questions about symptoms was similar to that of the Nordic questionnaire, which has been shown to have acceptable reliability [28].

Ensuring the accuracy with which the questionnaire was translated into local languages was a challenge. Care was taken to check the accuracy of translation by independent back-translation to English, and this revealed a number of problems. One was the distinction between “stairs” and “flights of stairs”, and despite attempts to resolve this problem, it is not certain that the term “30 flights of stairs” was always interpreted correctly. Therefore, this question will be ignored in future analyses based on the full dataset. Another difficulty arose with questions of the form “Do you expect that your back pain will be a problem in 12 months time”. In some languages this became “Do you expect your back pain will be a problem over the next 12 months”. Attempts were made to correct this misunderstanding, but it is possible that they were not fully successful.

In addition, terms such as “pain” may be understood differently in different languages even though translated as closely as possible. For this reason, when comparing countries, differences in the relative frequency of pain at different anatomical sites may be particularly revealing – there should have been little ambiguity in the understanding of anatomical sites since they were depicted clearly in diagrams. Interpretation should also be assisted by the questions that were asked about associated difficulty with tasks of daily living, since these were probably understood more uniformly.

Another difficulty that had not been expected was in the use of dates. It emerged that some participants in Iran and Japan used different numbering for calendar years, and where this occurred, corrections had to be made.

Some local investigators opted to include extra questions in addition to the core questions prescribed by CUPID. However, these additions were relatively minor and generally followed after the core questions. Thus, it seems unlikely that they will have influenced answers to the core questions importantly.

Ideally, all questionnaires would have been completed in the same way (interview or self-administration) by all participants. However, this proved impractical. Some occupational groups (especially manual workers in developing countries) would have had great difficulty in answering a written questionnaire, while some employers were unwilling to release their staff for interviews. Moreover, in New Zealand, where nurses and office workers were recruited from across the country, interviews would have been prohibitively expensive.

To explore whether the two methods of answering the questionnaire might lead to systematic differences in answers, we therefore elected to interview a random subset of UK participants while collecting data from the remainder by self-administration. Comparison of responses using the two approaches (Table 10) suggests that no major bias will have occurred as a consequence using both interviews and self-administration. However, if appropriate, method of data collection can be taken into account in statistical analyses.

Participation rates among subjects eligible for study were mostly high, but were less than 50% in five occupational groups (Table 2). We have no reason to expect that those who elected to take part were importantly unrepresentative in the prevalence of pain and its associations with risk factors. However, in future work it may be appropriate to carry out sensitivity analyses, excluding the occupational groups with the lowest response rates. The incomplete response to the baseline questionnaire will be less of a concern in longitudinal analyses based on the follow-up questionnaire.

The numbers of participants by occupational group that were suitable for analysis ranged from 92 to 1018 with a mean of 264. At the outset, our aim was to recruit at least 200 subjects in each group, and this was for the most part achieved (only 7 groups provided fewer than 150 subjects). Furthermore, the occupational groups studied varied substantially in their employment conditions (Table 3), access to healthcare (Table 4), and prevalence of psychosocial risk factors (Tables 7, 8, and 9). When exploring possible reasons for differences in the prevalence of pain and disability between occupational groups, it will be important to investigate these group-level characteristics as well as individual-level risk factors such as mental health and somatising tendency. The heterogeneity in their distribution should enhance statistical power to address their impact.

As might be expected, the demographic constitution of occupational groups also varied. In particular, many of the samples of nurses were largely or completely female, whereas some groups of “other workers” were all men. This reflects the nature of the occupations of interest. However, it should not be a major problem in interpretation of comparisons since there were an adequate number of occupational groups with a fairly even distribution of sex and age. Moreover, the occurrence of common musculoskeletal complaints appears not to vary greatly between men and women or between older and younger adults of working age [13], [23], [24].

In summary, the CUPID study is a major resource for the investigation of cultural and psychological determinants of common musculoskeletal disorders and associated disability. Although the data collected have inevitable limitations, the large differences in psychosocial risk factors (including knowledge and beliefs about MSDs) between occupational groups carrying out similar physical tasks in different countries should allow the study hypothesis to be addressed effectively. It will also allow exploration of differences in patterns of musculoskeletal complaint between the three categories of occupation examined, and the consistency of these differences across countries.

## Supporting Information

Appendix S1 **Committees which provided ethical approval for the cupid study.**
(DOCX)

Appendix S2 **Baseline questionnaire.**
(DOCX)

Appendix S3 **Follow-up questionnaire.**
(DOCX)
