# Correction: The CUPID (Cultural and Psychosocial Influences on Disability) Study: Methods of Data Collection and Characteristics of Study Sample

**DOI:** 10.1371/annotation/3faf76e5-f73e-427f-9d60-8f94939b0f7e

**Published:** 2012-10-11

**Authors:** David Coggon, Georgia Ntani, Keith T. Palmer, Vanda E. Felli, Raul Harari, Lope H. Barrero, Sarah A. Felknor, David Gimeno, Anna Cattrell, Consol Serra, Matteo Bonzini, Eleni Solidaki, Eda Merisalu, Rima R. Habib, Farideh Sadeghian, Masood Kadir, Sudath S. P. Warnakulasuriya, Ko Matsudaira, Busisiwe Nyantumbu, Malcolm R. Sim, Helen Harcombe, Ken Cox, Maria H. Marziale, Leila M. Sarquis, Florencia Harari, Rocio Freire, Natalia Harari, Magda V. Monroy, Leonardo A. Quintana, Marianela Rojas, Eduardo J. Salazar Vega, E. Clare Harris, Sergio Vargas-Prada, J. Miguel Martinez, George Delclos, Fernando G. Benavides, Michele Carugno, Marco M. Ferrario, Angela C. Pesatori, Leda Chatzi, Panos Bitsios, Manolis Kogevinas, Kristel Oha, Tuuli Sirk, Ali Sadeghian, Roshini J. Peiris-John, Nalini Sathiakumar, A. Rajitha Wickremasinghe, Noriko Yoshimura, Danuta Kielkowski, Helen L. Kelsall, Victor C. W. Hoe, Donna M. Urquhart, Sarah Derrett, David McBride, Andrew Gray

There was a typographical error in the antepenultimate author's name. The correct spelling is Sarah Derrett.

