# Supplementary material for: The CUPID (Cultural and Psychosocial Influences on Disability) Study: Methods of Data Collection and Characteristics of Study Sample
Source: PLoS One. 2012 Jul 6;7(7):e39820. doi: 10.1371/journal.pone.0039820 (PMC3391206; doi:10.1371/journal.pone.0039820)
Supplement: Appendix S2 — Baseline questionnaire. (DOCX) [file pone.0039820.s002.docx]

**APPENDIX S2**

**BASELINE QUESTIONNAIRE**

|  | **Serial Number** |
| --- | --- |

**INTERNATIONAL SURVEY OF WORK AND HEALTH**

| ***Please fill in the date that you complete this form*** | Date: | *day month year* |
| --- | --- | --- |

| **SECTION ONE: ABOUT YOURSELF** |
| --- |

| 1. | Please fill in your date of birth | | | | | | | | | | | | *day month year* | | | | | | | | | | | | |  |
| --- | --- | --- | --- | --- | --- | --- | --- | --- | --- | --- | --- | --- | --- | --- | --- | --- | --- | --- | --- | --- | --- | --- | --- | --- | --- | --- |
| 2. | *and* your sex | | | | | | | | | | | | Male  Female | | | | | | | | | | | | |  |
| 3. | Are you right or left-handed? | | | | | | | Right | | | |  | | Left | | |  | | | | Both equally | | | |  |  |
| 4. | How would you best describe your ethnic origin? **[Options will vary by country.]** | | | | | | | | | | | | | | | | | | | | | | | | |  |
| White British | | | |  | | | Bangladeshi | | | | | | | |  | | | | Indian | | | |  | | |  |
| Pakistani | | | |  | | | Black African/Caribbean | | | | | | | |  | | | | Chinese | | | |  | | |  |
| Other *(please specify)* | | | |  | |  | | | | | | | | | | | | | | | | | | | |  |
| 5. | | How old were you when you finished full time education? | | | | | | | | | | | | | | | | | | | | | | | | |
| Under 14 years | | |  | | | 14-16 years | | | |  | | 17-19 years | | | | |  | | 20 years or older | | | | | |  | |
| 6. | | How tall are you? | | cms or | | | | | | | ft  in | | | | | | | | | | | | | | | |
| 7a) | | Have you ever smoked regularly (ie at least once per day for a month or longer)? | | | | | | | | | | | | | | | | | | | No | |  | Yes |  | |
| b) | | If ***YES***, do you still smoke regularly? | | | | | | | | | | | | | | | | | | | No | |  | Yes |  | |

| **SECTION TWO: YOUR CURRENT WORK** |
| --- |

| 8. | What is your main occupation? | | | |  | | | | |  |  | | | |  |  |  |
| --- | --- | --- | --- | --- | --- | --- | --- | --- | --- | --- | --- | --- | --- | --- | --- | --- | --- |
| 9. | How long have you done this job? | | | | | | | | | | | | | | |  |  |
| Less than 1 year | | |  | 1-5 years | |  | More than 5 years | | | | |  | | | |  |  |
| 10. | | How many hours per week do you normally work in this job? | | | | | |  | | | | | Hours | | | | |
| 11. | | Does an **average working day** in the job involve any of the following?  *(Please tick* ***No*** *or* ***Yes*** *for each question)* | | | | | | |  | | | | | | | |  |
|  | | | | | | | | | *No* | | | | | *Yes* | | |  |
| a) Use of a keyboard or typewriter for more than four hours in total? | | | | | | | | |  | | | | |  | | |  |
| b) Other tasks involving repeated movements of the wrist or fingers for more than four hours in total? | | | | | | | | |  | | | | |  | | |  |
| c) Repeated bending and straightening of your elbow for longer than one hour in total? | | | | | | | | |  | | | | |  | | |  |
| d) Working for longer than one hour in total with your hands above shoulder height? | | | | | | | | |  | | | | |  | | |  |
| e) Lifting weights of 25 Kg (56 lbs) or more by hand? | | | | | | | | |  | | | | |  | | |  |
| f) Climbing up or down more than 30 flights of stairs a day? | | | | | | | | |  | | | | |  | | |  |
| g) Kneeling or squatting for longer than one hour in total? | | | | | | | | |  | | | | |  | | |  |
| h) Piecework in which you are paid according to the number of articles or tasks you or your team make or finish in the day? | | | | | | | | |  | | | | |  | | |  |
| i) A target number of articles or tasks that you or your team are expected to make or finish in the day? | | | | | | | | |  | | | | |  | | |  |
| j) Payment of a bonus if you make or finish more than an agreed number of articles/tasks in the day? | | | | | | | | |  | | | | |  | | |  |
| k) Working under pressure to complete tasks by a fixed time? | | | | | | | | |  | | | | |  | | |  |

| 12. | | In your job, do you have a choice in deciding: | | | | | | | | | | | | | | | | | | | | | | | | | | | | | | | | | | |
| --- | --- | --- | --- | --- | --- | --- | --- | --- | --- | --- | --- | --- | --- | --- | --- | --- | --- | --- | --- | --- | --- | --- | --- | --- | --- | --- | --- | --- | --- | --- | --- | --- | --- | --- | --- | --- |
|  | | | | | | | | | | |  | *Often* | | | | |  | *Sometimes* | | | | | | |  | *Seldom* | | | | | |  | *Never/*  *Almost Never* | | | |
| **How** you do your work? | | | | | | | | | | |  |  |  | | |  |  |  | |  | | | |  |  |  | |  | |  | |  |  |  | |  |
| **What** you do at work? | | | | | | | | | | |  |  |  | | |  |  |  | |  | | | |  |  |  | |  | |  | |  |  |  | |  |
| Your work timetable and breaks? | | | | | | | | | | |  |  |  | | |  |  |  | |  | | | |  |  |  | |  | |  | |  |  |  | |  |
| 13. | When you have difficulties in your work, how often do you get help and support from your colleagues or supervisor/manager? | | | | | | | | | | | | | | | | | | | | | | | | | | | | | | | | | | | |
| Often | | | |  | Sometimes | | |  | Seldom | | | | | | |  | | | Never | | | | | |  | | | | Not applicable | | | | | |  | |
| 14. | How satisfied have you been with your job as a whole, taking everything into consideration? | | | | | | | | | | | | | | | | | | | | | | | | | | | | | | | | | | | |
| Very satisfied | | | | | |  | Satisfied | | |  | | | | | Dissatisfied | | | | | | |  | | | | | Very dissatisfied | | | | | | | |  | |
| 15. | How secure do you feel your job would be if you had a significant illness that kept you off work for three months? | | | | | | | | | | | | | | | | | | | | | | | | | | | | | | | | | | | |
| Very safe | | | | | |  | Safe | | |  | | | | Rather unsafe | | | | | | | | |  | | | | Very unsafe | | | | | | | |  | |
| 16. Do you have any other job(s)? | | | | | | | | | | | | | | | | | | | | | No | | | | | | | | Yes | |  | | | |  | |
| If ***yes,*** what are your other job(s)? | | | | | | | | | | | | | | | | | | | | |  | | | | | | | |  | |  | | | |  | |
|  | | |  | | | | | | | | | | | | | | | | | |  | | | | | | | |  | |  | | | |  | |
|  | | |  | | | | | | | | | | | | | | | | | |  | | | | | | | |  | |  | | | |  | |

| **SECTION THREE: ACHES AND PAINS** |
| --- |

**LOW BACK PAIN IN PAST 12 MONTHS**

| 17a) | Have you had low back pain in the area shown below which lasted for more than a day at any time during the past 12 months? *(Do not include pain associated only with menstrual periods, pregnancy or during a course of a feverish illness.)* | | | | | |
| --- | --- | --- | --- | --- | --- | --- |
|  | 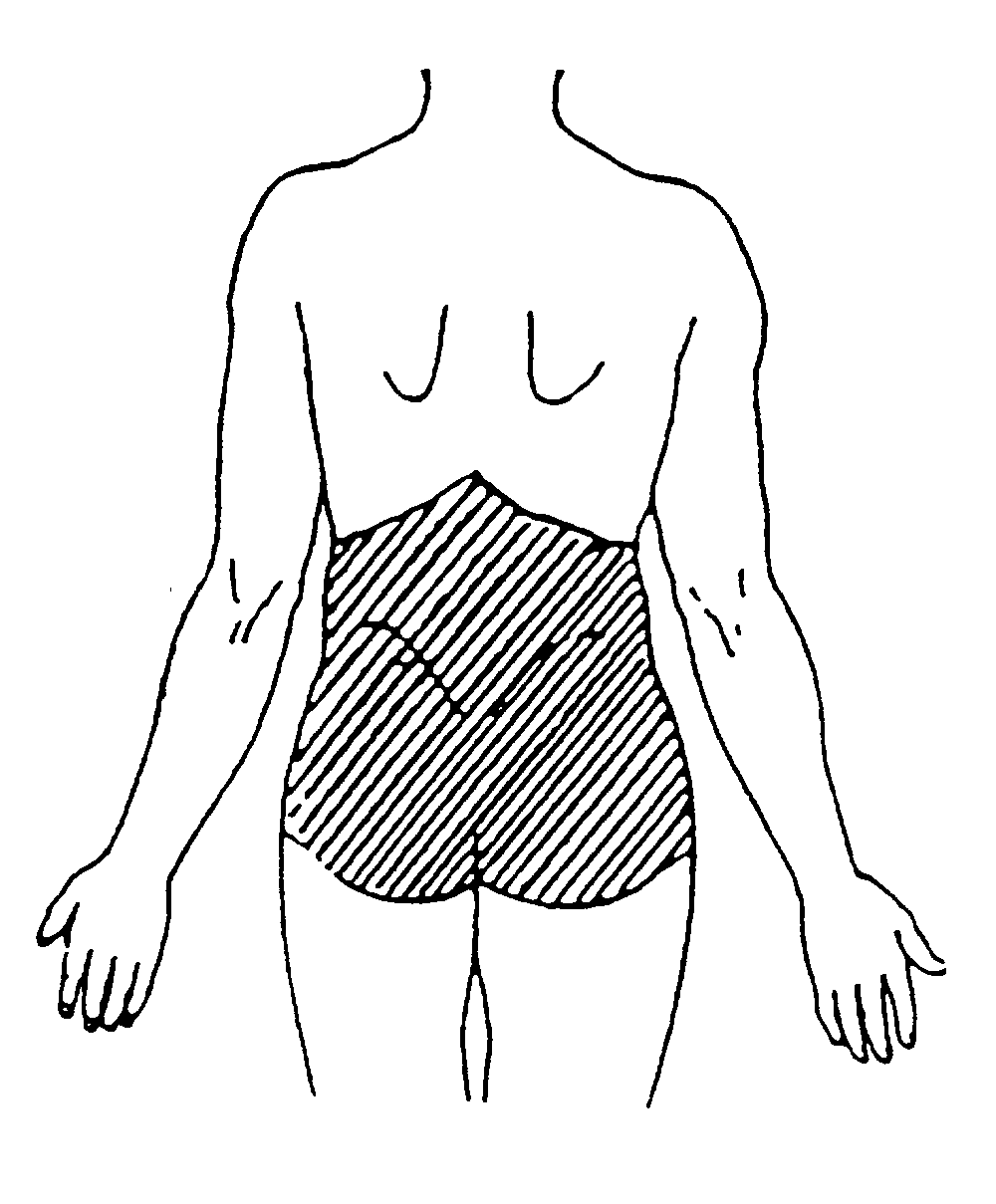 |  |  |  | |  |
|  |  | No |  | Yes |  |  |
|  |  | If ***NO*,** please go to question 22. If ***YES***, please continue. | | | | |

| b) | Within the past 12 months, has the pain ever spread down your leg(s) to below the knee (sciatica)? | | | | | | | | | | | No | |  | | Yes |  | | |
| --- | --- | --- | --- | --- | --- | --- | --- | --- | --- | --- | --- | --- | --- | --- | --- | --- | --- | --- | --- |
| c) | If you add together all the days on which you have had low back pain, during the past 12 months, how long a period would that make? | | | | | | | | | | | | | | | | | | |
|  | 1-6 days | | | |  | 1-4 weeks | | | | |  | 1-12 months | | | | |  | | |
| d) | Have you consulted a doctor or a medical person or alternative practitioner because of low back pain during the past 12 months? | | | | | | | | | | | No | |  | | Yes |  | | |
| e) | During the past 12 months on how many days did low back pain prevent you from going to work? | | | | | | | | | | | | | | | | | | |
|  | 0 days | |  | | 1-5 days | | |  | 6-30 days | | | |  | | More than 30 days | | | |  |
| 18. | | Do you expect that your back pain will be a problem in 12 months time? | | | | | | | | | | | | | | | | | |
|  | | No |  | Possibly | | |  | | | Probably | | |  | Definitely | | | |  | |

**LOW BACK PAIN IN PAST MONTH**

**We are particularly interested in any back pain you may have had during the past month**

| 19a) | Have you had low back pain in the area shown below which lasted for more than a day at any time during the past month? *(Do not include pain associated only with menstrual periods, pregnancy or during a course of a feverish illness.)* | | | | | | | | | | | | | | | | | | |
| --- | --- | --- | --- | --- | --- | --- | --- | --- | --- | --- | --- | --- | --- | --- | --- | --- | --- | --- | --- |
|  | 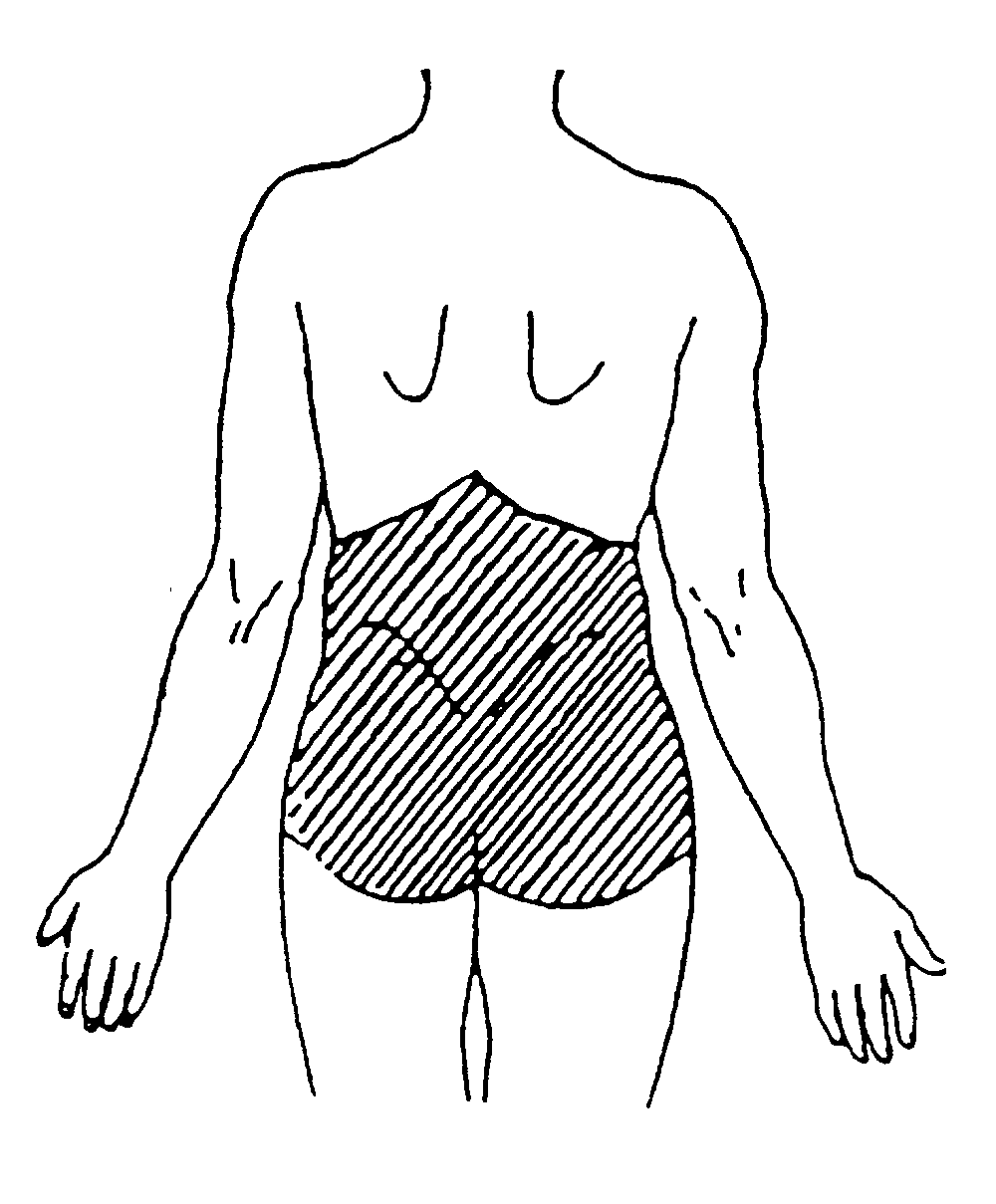 | | |  | | | | |  | |  | | | | |  | |  | |
|  |  |  |  | No | | | | |  | | Yes | | | | |  | |  | |
|  |  |  |  | If ***NO*,** please go to question 22. If ***YES***, please continue. | | | | | | | | | | | | | | | |
| b) | Within the past month, has the pain ever spread down your leg(s) to below the knee (sciatica)? | | | | | No | | |  | | Yes | | | | |  | | | |
| c) | If you add together all the days on which you have had low back pain, during the past month, how long a period would that make? | | | | | | | | | | | | | | | | | | |
|  | 1-6 days |  | 1-2 weeks | |  | | | More than 2 weeks | | | | | | | |  | | | |
| 20. | During the past month, has low back pain at any time made it difficult or impossible to do any of the following activities? | | | | | | | | | | | | | | | | | | |
|  | | | | | | | *No* | | |  | | *Difficult* | | | *Impossible* | | | | |
| a) | Cutting your toe nails | | | | | |  | | |  | | |  |  | | |  | |  |
| b) | Getting dressed | | | | | |  | | |  | | |  |  | | |  | |  |
| c) | Doing the jobs that you normally do around the house | | | | | |  | | |  | | |  |  | | |  | |  |
| 21. | Please think back to the last time that you were free from low back pain for a month or longer. When your most recent episode of low back pain then started, how did it begin? | | | | | | | | | | | | | | | | | | |
|  | Suddenly (ie within less than a minute) while you were at work | | | | | | | | | | | |  | | | | | | |
|  | Suddenly (ie within less than a minute) but not while you were at work | | | | | | | | | | | |  | | | | | | |
|  | Gradually | | | | | | | | | | | |  | | | | | | |

**NECK PAIN IN PAST 12 MONTHS**

| 22. | | Have you had pain in the neck in the area shown below which lasted for more than a day at any time during the past 12 months? | | | | | | | | | | | | | | | | | | | | | | | |
| --- | --- | --- | --- | --- | --- | --- | --- | --- | --- | --- | --- | --- | --- | --- | --- | --- | --- | --- | --- | --- | --- | --- | --- | --- | --- |
|  |  | | | | | | | | | | |  |  | |  | | | | | | |  | |  | |
|  |  |  |  |  |  |  |  |  |  |  |  | No |  | | Yes | | | | | | |  | |  | |
|  |  |  |  |  |  |  |  |  |  |  |  | If ***NO*,** please go to question 26. If ***YES***, please continue. | | | | | | | | | | | | | |
| b) | If you add together all the days on which you have had neck pain, in the past 12 months, how long a period would that make? | | | | | | | | | | | | | | | | | | | | | | | | |
|  | 1-6 days | | | | |  | 1-4 weeks | | | | | |  | | 1-12 months | | | | | | | |  | | |
| c) | Have you consulted a doctor or a medical person or alternative practitioner because of neck pain during the past 12 months? | | | | | | | | | | | | | | | | | No |  | Yes | | | | |  |
| d) | During the past 12 months on how many days did neck pain prevent you from going to work? | | | | | | | | | | | | | | | | | | | | | | | | |
|  | 0 days | |  | | 1-5 days | | | |  | 6-30 days | | | |  | | | More than 30 days | | | | | | | |  |
| 23. | Do you expect that your neck pain will be a problem in 12 months time? | | | | | | | | | | | | | | | | | | | | | | | | |
|  | No | |  | Possibly | | | |  | | | Probably | | |  | | Definitely | | | | |  | | | | |

**NECK PAIN IN THE PAST MONTH**

**We are particularly interested in any neck pain you may have had during the past month**

| 24a) | | Have you had pain in the neck in the area shown below which lasted for more than a day at any time during the past month? | | | | | | | | | | | | | | | | | | | | | |
| --- | --- | --- | --- | --- | --- | --- | --- | --- | --- | --- | --- | --- | --- | --- | --- | --- | --- | --- | --- | --- | --- | --- | --- |
|  | |  | | | |  | |  | | | |  | | | | | | | | |  | |  |
|  | |  |  |  |  | No | |  | | | | Yes | | | | | | | | |  | |  |
|  | |  |  |  |  | If ***NO*,** please go to question 26. If ***YES***, please continue. | | | | | | | | | | | | | | | | | |
| b) | | If you add together all the days on which you have had neck pain, during the past month, how long a period would that make? | | | | | | | | | | | | | | | | | | | | | |
|  | 1-6 days | |  | 1-2 weeks |  | | | | | More than 2 weeks | | | | | | | |  | | | | | |
| 25. | | During the past month, has neck pain at any time made it difficult or impossible to do any of the following activities? | | | | | | | | | | | | | | | | | | | | | |
|  | | | | | | |  | | *No* | | | | |  | *Difficult* | | | |  | *Impossible* | | | |
| a) Getting dressed | | | | | | |  | |  | |  | |  |  |  |  |  | |  |  | |  | |
| b) Doing the jobs that you normally do around the house | | | | | | | | | | |  | |  |  |  |  |  | |  |  | |  | |

**SHOULDER PAIN IN PAST 12 MONTHS**

| 26a) | | Have you had pain in the shoulder in the area shown below which lasted for more than a day at any time during the past 12 months? | | | | | | | | | | | | | | | | | | | | | |
| --- | --- | --- | --- | --- | --- | --- | --- | --- | --- | --- | --- | --- | --- | --- | --- | --- | --- | --- | --- | --- | --- | --- | --- |
|  | | | | | | | | | No | | | | | | Right shoulder only | | | | | | | | |
|  |  |  |  |  |  |  |  |  | Left shoulder only | | | | | | Both shoulders | | | | | | | | |
|  | | | | | | | | | If ***NO***, please go to question 30. If ***YES*** please continue. | | | | | | | | | | | | | | |
| b) | If you add together all the days on which you have had shoulder pain, in the past 12 months, how long a period would that make? | | | | | | | | | | | | | | | | | | | | | |  |
|  | 1-6 days | | |  | 1-4 weeks | | | | | |  | 1-12 months | | | | | | |  | | | |  |
| c) | Have you consulted a doctor or a medical person or alternative practitioner because of shoulder pain during the past 12 months? | | | | | | | | | | | | | | | | No |  | | Yes | |  |  |
| d) | During the past 12 months on how many days did shoulder pain prevent you from going to work? | | | | | | | | | | | | | | | | | | | | | |  |
|  | 0 days |  | | 1-5 days | | |  | | 6-30 days | | | |  | | | More than 30 days | | | | | |  |  |
| 27. | Do you expect that your shoulder pain will be a problem in 12 months time? | | | | | | | | | | | | | | | | | | | | | |  |
|  | No |  | Possibly | | |  | | | | Probably | | |  | | Definitely | | | | | |  | |  |

**SHOULDER PAIN IN THE PAST MONTH**

**We are particularly interested in any shoulder pain you may have had during the past month**

| 28. | | Have you had pain in the shoulder in the area shown below which lasted for more than a day at any time during the past month? | | | | | | | | | | | | | | |
| --- | --- | --- | --- | --- | --- | --- | --- | --- | --- | --- | --- | --- | --- | --- | --- | --- |
|  | | | | | No | | | | Right shoulder only | | | | | | | |
|  |  |  |  |  | Left shoulder only | | | | Both shoulders | | | | | | | |
|  | | | | | If ***NO***, please go to question 30. If ***YES*** please continue. | | | | | | | | | | | |
| b) | If you add together all the days on which you have had shoulder pain, during the past month, how long a period would that make? | | | | | | | | | | | | | | |  |
|  | 1-6 days |  | 1-2 weeks | |  | More than 2 weeks | | | | |  | | | | |  |
| 29. | During the past month, has shoulder pain at any time made it difficult or impossible to do any of the following activities? | | | | | | | | | | | | | | |  |
|  | | | | |  | | *No* | | *Difficult* | | | | *Impossible* | | |  |
| a) Combing or brushing your hair | | | | | | |  | |  |  | |  | |  |  |  |
| b) Bathing/Showering | | | | | | |  | |  |  | |  | |  |  |  |
| c) Getting dressed | | | | | | |  | |  |  | |  | |  |  |  |
| d) Doing the jobs that you normally do around the house | | | | | | |  | |  |  | |  | |  |  |  |

**ELBOW PAIN IN THE PAST 12 MONTHS**

| 30. | Have you had pain in the elbow in the area shown below which lasted for more than a day at any time during the past 12 months? | | | | | | | | | | | | | | | | | | | | | | | |  |
| --- | --- | --- | --- | --- | --- | --- | --- | --- | --- | --- | --- | --- | --- | --- | --- | --- | --- | --- | --- | --- | --- | --- | --- | --- | --- |
|  | | | | | | | | | | | No | | | | | | | Right elbow only | | | | | | | |
|  |  |  |  |  |  |  |  |  |  |  | Left elbow only | | | | | | | Both elbows | | | | | | | |
|  | | | | | | | | | | | If ***NO,*** please go to question 34. If ***YES*** please continue. | | | | | | | | | | | | | | |
| b) | If you add together all the days on which you have had elbow pain, in the past 12 months, how long a period would that make? | | | | | | | | | | | | | | | | | | | | | | | |  |
|  | 1-6 days | | | |  | | 1-4 weeks | | | | | |  | 1-12 months | | | | | | | |  | | |  |
| c) | Have you consulted a doctor or a medical person or alternative practitioner because of elbow pain during the past 12 months? | | | | | | | | | | | | | | | | | | | No | Yes | | | |  |
| d) | During the past 12 months on how many days did elbow pain prevent you from going to work? | | | | | | | | | | | | | | | | | | | | | | | |  |
|  | 0 days | |  | | | 1-5 days | | |  | | 6-30 days | | | | |  | | | More than 30 days | | | | |  |  |
| 31. | | Do you expect that your elbow pain will be a problem in 12 months time? | | | | | | | | | | | | | | | | | | | | | | |  |
|  | | No |  | Possibly | | | |  | | | | Probably | | |  | | | Definitely | | | | |  | |  |

**ELBOW PAIN IN THE PAST MONTH**

**We are particularly interested in any elbow pain you may have had during the past month**

| 32. | Have you had pain in the elbow in the area shown below which lasted for more than a day at any time during the past month? | | | | | | | | | | | | | | | | | | |  |
| --- | --- | --- | --- | --- | --- | --- | --- | --- | --- | --- | --- | --- | --- | --- | --- | --- | --- | --- | --- | --- |
|  | | | | | | No | | | | | | Right elbow only | | | | | | | | |
|  |  |  |  |  |  | Left elbow only | | | | | | Both elbows | | | | | | | | |
|  | | | | | | If ***NO,*** please go to question 34. If ***YES*** please continue. | | | | | | | | | | | | | | |
| b) | | If you add together all the days on which you have had elbow pain, during the past month, how long a period would that make? | | | | | | | | | | | | | | | | | |  |
|  | | 1-6 days |  | 1-2 weeks | | |  | | More than 2 weeks | | | | | |  | | | | |  |
| 33. | | During the past month, has elbow pain at any time made it difficult or impossible to do any of the following activities? | | | | | | | | | | | | | | | | | |  |
|  | | | | | |  | |  | | *No* | |  | *Difficult* | | | |  | *Impossible* | |  |
| a) Opening bottles, jars or taps | | | | | | | | | |  | |  |  |  | |  |  |  |  |  |
| b) Getting dressed | | | | | |  | |  | |  | |  |  |  | |  |  |  |  |  |
| c) Doing the jobs that you normally do around the house | | | | | | | | | |  | |  |  |  | |  |  |  |  |  |

**WRIST AND HAND PAIN IN PAST 12 MONTHS**

| 34. | Have you had pain in the wrist or hand in the area shown below which lasted for more than a day at any time during the past 12 months? | | | | | | | | | | | | | | | | | | | | | | | |  |
| --- | --- | --- | --- | --- | --- | --- | --- | --- | --- | --- | --- | --- | --- | --- | --- | --- | --- | --- | --- | --- | --- | --- | --- | --- | --- |
|  | | | | | | | | No | | | | | | | | | | Right hand or wrist only | | | | | | | |
|  |  |  |  |  |  |  |  | Left hand or wrist only | | | | | | | | | | Both hands or wrists | | | | | | | |
|  | | | | | | | | If ***NO,*** please go to question 38. If ***YES*** please continue. | | | | | | | | | | | | | | | | | |
| b) | If you add together all the days on which you have had wrist/hand pain, in the past 12 months, how long a period would that make? | | | | | | | | | | | | | | | | | | | | | | | |  |
|  | 1-6 days | | | |  | | | 1-4 weeks | | | | |  | 1-12 months | | | | | | | |  | | |  |
| c) | Have you consulted a doctor or a medical person or alternative practitioner because of wrist/hand pain during the past 12 months? | | | | | | | | | | | | | | | | | | | No | Yes | | | |  |
| d) | During the past 12 months on how many days did wrist/hand pain prevent you from going to work? | | | | | | | | | | | | | | | | | | | | | | | |  |
|  | 0 days | |  | | | 1-5 days | | | |  | 6-30 days | | | | |  | | | More than 30 days | | | | |  |  |
| 35. | | Do you expect that your wrist/hand pain will be a problem in 12 months time? | | | | | | | | | | | | | | | | | | | | | | |  |
|  | | No |  | Possibly | | | | |  | | | Probably | | |  | | | Definitely | | | | |  | |  |

**WRIST AND HAND PAIN IN THE PAST MONTH**

**We are particularly interested in any wrist/hand pain you may have had during the past month**

| 36. | Have you had pain in the wrist or hand in the area shown below which lasted for more than a day at any time during the past month? | | | | | | | | | | | | |  |
| --- | --- | --- | --- | --- | --- | --- | --- | --- | --- | --- | --- | --- | --- | --- |
|  | | | | | | No | | | | | Right hand or wrist only | | | |
|  |  |  |  |  |  | Left hand or wrist only | | | | | Both hands or wrists | | | |
|  | | | | | | If ***NO,*** please go to question 38. If ***YES*** please continue. | | | | | | | | |
| b) | If you add together all the days on which you have had wrist/hand pain, during the past month, how long a period would that make? | | | | | | | | | | | | |  |
|  | | 1-6 days |  | 1-2 weeks | | |  | More than 2 weeks | | | |  | |  |
| 37. | During the past month, has wrist/hand pain at any time made it difficult or impossible to do any of the following activities? | | | | | | | | | | | | |  |
|  | | | | | |  | | | *No* | | *Difficult* | | *Impossible* | |
| a) Writing | | | | | | | | |  | |  | |  | |
| b) Locking and unlocking doors | | | | | | | | |  | |  | |  | |
| c) Opening bottles, jars or taps | | | | | | | | |  | |  | |  | |
| d) Getting dressed | | | | | | | | |  | |  | |  | |
| e) Doing the jobs that you normally do around the house | | | | | | | | |  | |  | |  | |

**KNEE PAIN IN THE PAST 12 MONTHS**

| 38. | Have you had pain in the knee in the area shown below which lasted for more than a day at any time during the past 12 months? | | | | | | | | | | | | | | | | | | | | | | | |  |
| --- | --- | --- | --- | --- | --- | --- | --- | --- | --- | --- | --- | --- | --- | --- | --- | --- | --- | --- | --- | --- | --- | --- | --- | --- | --- |
| 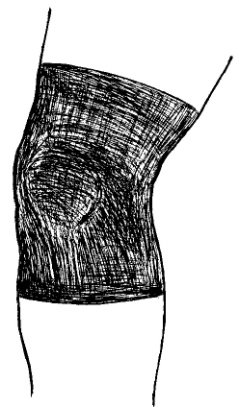 | | | | | | | | | | No | | | | | | | | | Right knee only | | | | | | |
|  |  |  |  |  |  |  |  |  |  | Left knee only | | | | | | | | | Both knees | | | | | | |
|  | | | | | | | | | | If ***NO,*** please go to question 42. If ***YES*** please continue. | | | | | | | | | | | | | | | |
| b) | If you add together all the days on which you have had knee pain, in the past 12 months, how long a period would that make? | | | | | | | | | | | | | | | | | | | | | | | |  |
|  | 1-6 days | | |  | | 1-4 weeks | | | | | |  | 1-12 months | | | | | | | |  | | | |  |
| c) | Have you consulted a doctor or a medical person or alternative practitioner because of knee pain during the past 12 months? | | | | | | | | | | | | | | | | | | No |  | Yes | |  | |  |
| d) | During the past 12 months on how many days did knee pain prevent you from going to work? | | | | | | | | | | | | | | | | | | | | | | | |  |
|  | 0 days |  | | | 1-5 days | | |  | | 6-30 days | | | | |  | | More than 30 days | | | | | | |  |  |
| 39. | Do you expect that your knee pain will be a problem in 12 months time? | | | | | | | | | | | | | | | | | | | | | | | |  |
|  | No |  | Possibly | | | |  | | | | Probably | | |  | | Definitely | | | | | |  | | |  |

**KNEE PAIN IN THE PAST MONTH**

**We are particularly interested in any knee pain you may have had during the past month**

| 40. | Have you had pain in the knee in the area shown below which lasted for more than a day at any time during the past month? | | | | | | | | | | | |  |
| --- | --- | --- | --- | --- | --- | --- | --- | --- | --- | --- | --- | --- | --- |
| 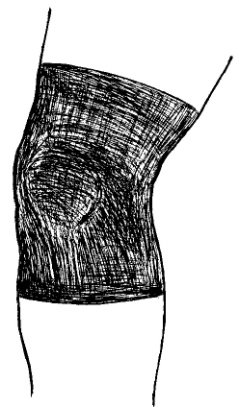 | | | | | No | | | | | | Right knee only | | |
|  |  |  |  |  | Left knee only | | | | | | Both knees | | |
|  | | | | | If ***NO,*** please go to question 42. If ***YES*** please continue. | | | | | | | | |
| b) | If you add together all the days on which you have had knee pain, during the past month, how long a period would that make? | | | | | | | | | | | |  |
|  | 1-6 days |  | 1-2 weeks | | |  | More than 2 weeks | | | |  | |  |
| 41. | During the past month, has knee pain at any time made it difficult or impossible to do any of the following activities? | | | | | | | | | | | |  |
|  | | | | |  | | | *No* | *Difficult* | | | *Impossible* |  |
| a) Walking up and down stairs | | | | | | | |  |  | | |  |  |
| b) Walking on level ground | | | | | | | |  |  | | |  |  |
| c) Getting dressed | | | | | | | |  |  | | |  |  |
| d) Doing the jobs that you normally do around the house | | | | | | | |  |  | | |  |  |

| **SECTION FOUR: OTHER PEOPLE’S PAIN** |
| --- |

**LOW BACK PAIN**

| 42. | Do you know anyone who has had low back pain in the past 12 months? | | | | | |
| --- | --- | --- | --- | --- | --- | --- |
|  | a) | At work | No |  | Yes |  |
|  | b) | Outside work | No |  | Yes |  |

**NECK PAIN**

| 43. | | Do you know anyone who has had neck pain in the past 12 months? | | | | | |
| --- | --- | --- | --- | --- | --- | --- | --- |
|  | a) | | At work | No |  | Yes |  |
|  | b) | | Outside work | No |  | Yes |  |

**PAIN IN THE ARM, SHOULDER OR HAND**

| 44. | Do you know anyone who has had pain in the arm, shoulder or hand in the past 12 months? | | | | | | |
| --- | --- | --- | --- | --- | --- | --- | --- |
|  | | a) | At work | No |  | Yes |  |
|  | | b) | Outside work | No |  | Yes |  |

**KNEE PAIN**

| 45. | Do you know anyone who has had knee pain in the past 12 months? | | | | | | |
| --- | --- | --- | --- | --- | --- | --- | --- |
|  | | a) | At work | No |  | Yes |  |
|  | | b) | Outside work | No |  | Yes |  |

| **SECTION FIVE: YOUR VIEWS ON THE CAUSES AND PREVENTION OF PAIN** |
| --- |

| 46. | Based on your own views and what the doctor or others may have told you about pain in the arm, shoulder or hands, how strongly do you agree with the following statements?  *(Tick one box on each line.)* | | | | | | | | | | | | | | | | | | | | | | | | | |
| --- | --- | --- | --- | --- | --- | --- | --- | --- | --- | --- | --- | --- | --- | --- | --- | --- | --- | --- | --- | --- | --- | --- | --- | --- | --- | --- |
| *For someone with this problem ..* | |  | *Completely disagree* | | | | | | |  | *Tend to disagree* | | |  | *Unsure* | | | |  | *Tend to agree* |  | *Completely agree* | | |  |  |
| Physical activity should be avoided as it might harm the arm | | |  | |  | | |  | |  |  |  |  |  |  |  | |  |  |  |  |  | | |  |  |
| These problems usually get better within three months | |  |  | |  | | |  | |  |  |  |  |  |  |  | |  |  |  |  |  | | |  |  |
| Rest is needed to get better | |  |  | |  | | |  | |  |  |  |  |  |  |  | |  |  |  |  |  | | |  |  |
| Neglecting problems of this kind can cause permanent health problems | |  |  | |  | | |  | |  |  |  |  |  |  |  | |  |  |  |  |  | | |  |  |
| These problems are commonly caused by people’s work | |  |  | |  | | |  | |  |  |  |  |  |  |  | |  |  |  |  |  | | |  |  |
| 47. | Based on your own views and what the doctor or others may have told you about low-back pain, how strongly do you agree with the following statements? *(Tick one box on each line.)* | | | | | | | | | | | | | | | | | | | | | | | | | |
| *For someone with this problem ..* | |  | *Completely disagree* | | | | | | |  | *Tend to disagree* | | |  | *Unsure* | | | |  | *Tend to agree* |  | *Completely agree* | | |  |  |
| Physical activity should be avoided as it might harm the back | | |  | | |  | | |  |  |  |  |  |  |  |  | |  |  |  |  |  |  |  |  |  |
| These problems usually get better within three months | | |  | | |  | | |  |  |  |  |  |  |  |  | |  |  |  |  |  |  |  |  |  |
| Rest is needed to get better | |  |  | | |  | | |  |  |  |  |  |  |  |  | |  |  |  |  |  |  |  |  |  |
| Neglecting problems of this kind can cause permanent health problems | | |  | | |  | | |  |  |  |  |  |  |  |  | |  |  |  |  |  |  |  |  |  |
| These problems are commonly caused by people’s work | | |  | | |  | | |  |  |  |  |  |  |  |  | |  |  |  |  |  |  |  |  |  |
|  |  |  |  |  |  |  |  |  |  |  |  |  |  |  |  |  |  |  |  |  |  |  |  |  |  |  |
| 48. | Have you ever heard or read about repetitive strain injury (RSI), work related upper limb disorder (WRULD) or cumulative trauma syndrome (CTS)? **[Appropriate terms may vary by country.]** | | | | | | | | | | | | | | | | | | | | | | | | |  |
| No | | | |  | | | Yes | | | | | | | |  | |  | | | | | | | | |  |

| **SECTION SIX: YOUR HEALTH MORE GENERALLY** |
| --- |

**PAST 7 DAYS**

| 49. | Below is a list of problems people sometimes have. Please read each one carefully and circle the number that best describes HOW MUCH THAT PROBLEM HAS DISTRESSED OR BOTHERED YOU DURING THE **PAST 7 DAYS INCLUDING TODAY**  *Circle only* ***one number*** *for each problem and do not skip any items* | | | | | | | | | | | | | | | | | | | | | |
| --- | --- | --- | --- | --- | --- | --- | --- | --- | --- | --- | --- | --- | --- | --- | --- | --- | --- | --- | --- | --- | --- | --- |
|  | |  | *Not at all* | | |  | *A little bit* | | |  | *Moderately* | | |  | *Quite a bit* | | |  | *Extremely* | | |  |
| a) Faintness or dizziness | |  |  | 0 |  |  |  | 1 |  |  |  | 2 |  |  |  | 3 |  |  |  | 4 |  |  |
| b) Pains in the heart or chest | |  |  | 0 |  |  |  | 1 |  |  |  | 2 |  |  |  | 3 |  |  |  | 4 |  |  |
| c) Nausea or upset stomach | |  |  | 0 |  |  |  | 1 |  |  |  | 2 |  |  |  | 3 |  |  |  | 4 |  |  |
| d) Trouble getting your breath | |  |  | 0 |  |  |  | 1 |  |  |  | 2 |  |  |  | 3 |  |  |  | 4 |  |  |
| e) Numbness or tingling in parts of your body | |  |  | 0 |  |  |  | 1 |  |  |  | 2 |  |  |  | 3 |  |  |  | 4 |  |  |
| f) Feeling weak in parts of your body | |  |  | 0 |  |  |  | 1 |  |  |  | 2 |  |  |  | 3 |  |  |  | 4 |  |  |
| g) Hot or cold spells | |  |  | 0 |  |  |  | 1 |  |  |  | 2 |  |  |  | 3 |  |  |  | 4 |  |  |

**PAST MONTH**

| 50. | These questions are about how you feel and how things have been with you **during the past month.** For each question, please give the one answer that best describes how things have been for you during the past month. How much of the time during the **past month**:  *Circle* ***one number*** *on each line* | | | | | | |  |
| --- | --- | --- | --- | --- | --- | --- | --- | --- |
|  | | ***All of the time*** | ***Most of the time*** | ***A good bit of the time*** | ***Some of the time*** | ***A little of the time*** | ***None of the time*** | |
| a) Were you a happy person? | | 1 | 2 | 3 | 4 | 5 | 6 | |
| b) Have you felt calm and peaceful? | | 1 | 2 | 3 | 4 | 5 | 6 | |
| c) Have you been a very nervous person | | 1 | 2 | 3 | 4 | 5 | 6 | |
| d) Have you felt downhearted and low? | | 1 | 2 | 3 | 4 | 5 | 6 | |
| e) Have you felt so down that nothing could cheer you up? | | 1 | 2 | 3 | 4 | 5 | 6 | |

**PAST 12 MONTHS**

| 51. | Over the past 12 months, on how many days in total have you been prevented from going to work because of | | | | | | | | |
| --- | --- | --- | --- | --- | --- | --- | --- | --- | --- |
| a) a problem with your back, neck, shoulder, elbow, wrist, hand or knees | | | | | | | | | |
|  | | 0 days |  | 1-5 days |  | 6-30 days |  | More than 30 days |  |
| b) other illness | | | | | | | | | |
|  | | 0 days |  | 1-5 days |  | 6-30 days |  | More than 30 days |  |
