# Supplementary material for: The CUPID (Cultural and Psychosocial Influences on Disability) Study: Methods of Data Collection and Characteristics of Study Sample
Source: PLoS One. 2012 Jul 6;7(7):e39820. doi: 10.1371/journal.pone.0039820 (PMC3391206; doi:10.1371/journal.pone.0039820)
Supplement: Appendix S3 — Follow-up questionnaire. (DOCX) [file pone.0039820.s003.docx]

**APPENDIX S3**

**FOLLOW-UP QUESTIONNAIRE**

|  | **Serial Number** |
| --- | --- |

**INTERNATIONAL SURVEY OF WORK AND HEALTH**

| ***Please fill in the date that you complete this form*** | Date: | *day month year* |
| --- | --- | --- |

| **SECTION ONE: ABOUT YOURSELF** |
| --- |

| 1. | Please fill in your date of birth | *day month year* |
| --- | --- | --- |
| 2. | *and* your sex | Male  Female |

| **SECTION TWO: YOUR CURRENT WORK** |
| --- |

| 3a) | Do you still have the same main job as when we last questioned you about a year ago? | No |  | Yes | |  |
| --- | --- | --- | --- | --- | --- | --- |
| If ***YES,*** please go to question 4. If ***NO,*** please continue | | | | | | |
| b) | Did you leave that job because of medical problems with your back, neck, shoulder, elbow, wrist, hand or knee? | No |  | | Yes |  |
| c) | And do you have another job now? | No |  | | Yes |  |

| **SECTION THREE: ACHES AND PAINS** |
| --- |

**LOW BACK PAIN IN PAST MONTH**

| 4a) Have you had low back pain in the area shown below which lasted for more than a day at any time during the past month? *(Do not include pain associated only with menstrual periods, pregnancy or during a course of a feverish illness.)* | | | | | | |
| --- | --- | --- | --- | --- | --- | --- |
|  | 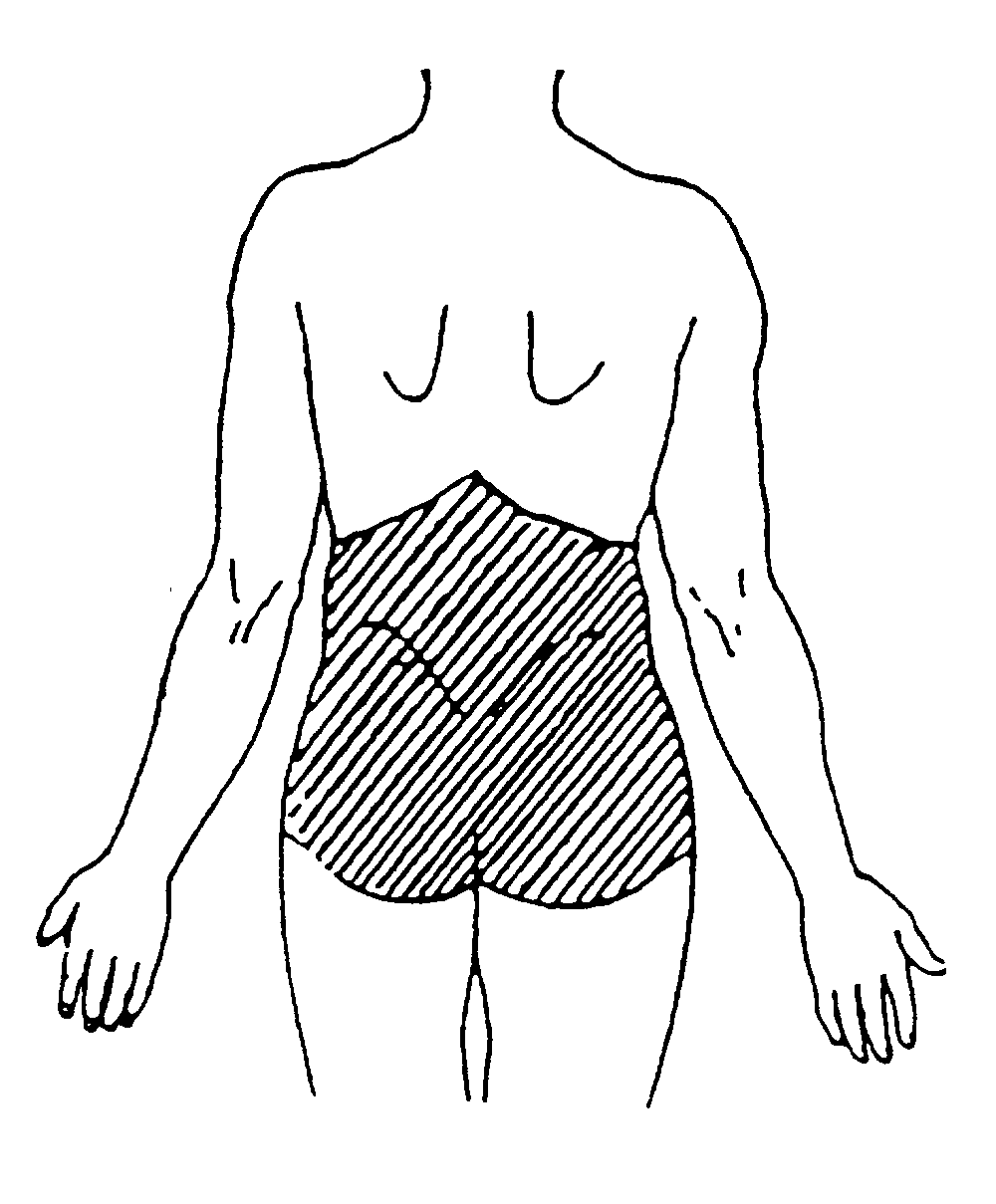 |  |  |  | |  |
|  |  | No |  | Yes |  |  |
|  |  | If ***NO*,** please go to question 8. If ***YES***, please continue. | | | | |

| b) | If you add together all the days on which you have had low back pain, during the past month, how long a period would that make? | | | | | | |
| --- | --- | --- | --- | --- | --- | --- | --- |
|  | | 1-6 days |  | 1-2 weeks |  | More than 2 weeks |  |

| 5. | During the past month, has low back pain at any time made it difficult or impossible to do any of the following activities? | | | | | | | | | | | | | | |
| --- | --- | --- | --- | --- | --- | --- | --- | --- | --- | --- | --- | --- | --- | --- | --- |
|  | | | | |  | *No* | | | | *Difficult* | | | *Impossible* | | |
| a) Cutting your toe nails | | | | | |  | | | |  | | |  | | |
| b) Getting dressed | | | | | |  | | | |  | | |  | | |
| c) Doing the jobs that you normally do around the house | | | | | |  | | | |  | | |  | | |
| 6. | During the past month on how many days has low back pain prevented you from going to work? | | | | | | | | | | | | | | |
|  | 0 days |  | 1-5 days |  | More than 5 days | | |  | Not applicable because unemployed | | | | | |  |
| 7. | Within the past month, has the pain ever spread down your leg(s) to below the knee (sciatica)? | | | | | | No | | | |  | Yes | |  | |

**NECK PAIN IN THE PAST MONTH**

| 8a) | Have you had pain in the neck in the area shown below which lasted for more than a day at any time during the past month? | | | | | | | | | | | | | | | | | | | | | | |
| --- | --- | --- | --- | --- | --- | --- | --- | --- | --- | --- | --- | --- | --- | --- | --- | --- | --- | --- | --- | --- | --- | --- | --- |
|  |  | | | | | | | | | |  |  | | | |  | | | | |  | |  |
|  |  |  |  |  |  |  |  |  |  |  | No |  | | | | Yes | | | | |  | |  |
|  |  |  |  |  |  |  |  |  |  |  | If ***NO*,** please go to question 11. If ***YES***, please continue. | | | | | | | | | | | | |
| b) | If you add together all the days on which you have had neck pain, during the past month, how long a period would that make? | | | | | | | | | | | | | | | | | | | | | | |
|  | | 1-6 days | | |  | 1-2 weeks | | | |  | | | More than 2 weeks | | | | | |  | | | | |
| 9. | During the past month, has neck pain at any time made it difficult or impossible to do any of the following activities? | | | | | | | | | | | | | | | | | | | | | | |
|  | | | | | | | |  | | | | | | *No* | | | | *Difficult* | | *Impossible* | | | |
| a) Getting dressed | | | | | | | | | | | | | |  | | | |  | |  | | | |
| b) Doing the jobs that you normally do around the house | | | | | | | | | | | | | |  | | | |  | |  | | | |
| 10. During the past month on how many days has neck pain prevented you from going to work? | | | | | | | | | | | | | | | | | | | | | | | |
|  | | 0 days |  | 1-5 days | | |  | | More than 5 days | | | | | |  | | Not applicable because unemployed | | | | |  | |

**SHOULDER PAIN IN THE PAST month**

| 11a) | | Have you had pain in the shoulder in the area shown below which lasted for more than a day at any time during the past month? | | | | | | | | | | | | | | | | | |
| --- | --- | --- | --- | --- | --- | --- | --- | --- | --- | --- | --- | --- | --- | --- | --- | --- | --- | --- | --- |
|  | | | | | | | | No | | | | | | Right shoulder only | | | | | |
|  |  |  |  |  |  |  |  | Left shoulder only | | | | | | Both shoulders | | | | | |
|  | | | | | | | | If ***NO***, please go to question 14. If ***YES*** please continue. | | | | | | | | | | | |
| b) | If you add together all the days on which you have had shoulder pain, during the past month, how long a period would that make? | | | | | | | | | | | | | | | | | |  |
|  | 1-6 days | | |  | 1-2 weeks | | | |  | More than 2 weeks | | | | | |  | | |  |
| 12. | During the past month, has shoulder pain at any time made it difficult or impossible to do any of the following activities? | | | | | | | | | | | | | | | | | |  |
|  | | | | | | | |  | | | *No* | | | | *Difficult* | | *Impossible* | |  |
| a) Combing or brushing your hair | | | | | | | | | | |  | | | |  | |  | |  |
| b) Bathing/showering | | | | | | | | | | |  | | | |  | |  | |  |
| c) Getting dressed | | | | | | | | | | |  | | | |  | |  | |  |
| d) Doing the jobs that you normally do around the house | | | | | | | | | | |  | | | |  | |  | |  |
| 13. | During the past month on how many days has shoulder pain prevented you from going to work? | | | | | | | | | | | | | | | | | |  |
|  | 0 days |  | 1-5 days | | |  | | More than 5 days | | | |  | | Not applicable because unemployed | | | |  |  |

**ELBOW PAIN IN THE PAST MONTH**

| 14a) | Have you had pain in the elbow in the area shown below which lasted for more than a day at any time during the past month? | | | | | | | | | | | | | | | | | | | |  |
| --- | --- | --- | --- | --- | --- | --- | --- | --- | --- | --- | --- | --- | --- | --- | --- | --- | --- | --- | --- | --- | --- |
|  | | | | | | | | | No | | | | | | | Right elbow only | | | | | |
|  |  |  |  |  |  |  |  |  | Left elbow only | | | | | | | Both elbows | | | | | |
|  | | | | | | | | | If ***NO,*** please go to question 17. If ***YES*** please continue. | | | | | | | | | | | | |
| b) | If you add together all the days on which you have had elbow pain, during the past month, how long a period would that make? | | | | | | | | | | | | | | | | | | | |  |
|  | 1-6 days | | | |  | 1-2 weeks | | | | |  | More than 2 weeks | | | | | |  | | |  |
| 15. | During the past month, has elbow pain at any time made it difficult or impossible to do any of the following activities? | | | | | | | | | | | | | | | | | | | |  |
|  | | | | | | | | |  | | | | *No* | | | | *Difficult* | | *Impossible* | |  |
| a) Opening bottles, jars or taps | | | | | | | | | | | | |  | | | |  | |  | |  |
| b) Getting dressed | | | | | | | | | | | | |  | | | |  | |  | |  |
| c) Doing the jobs that you normally do around the house | | | | | | | | | | | | |  | | | |  | |  | |  |
| 16. During the past month on how many days has elbow pain prevented you from going to work? | | | | | | | | | | | | | | | | | | | | |  |
|  | | 0 days |  | 1-5 days | | |  | | | More than 5 days | | | |  | | Not applicable because unemployed | | | |  |  |

**WRIST AND HAND PAIN IN THE PAST MONTH**

| 17a) | Have you had pain in the wrist or hand in the area shown below which lasted for more than a day at any time during the past month? | | | | | | | | | | | | | | | | | | |  |
| --- | --- | --- | --- | --- | --- | --- | --- | --- | --- | --- | --- | --- | --- | --- | --- | --- | --- | --- | --- | --- |
|  | | | | | | | | No | | | | | | | Right hand or wrist only | | | | | |
|  |  |  |  |  |  |  |  | Left hand or wrist only | | | | | | | Both hands or wrists | | | | | |
|  | | | | | | | | If ***NO,*** please go to question 20. If ***YES*** please continue. | | | | | | | | | | | | |
| b) | If you add together all the days on which you have had wrist/hand pain, during the past month, how long a period would that make? | | | | | | | | | | | | | | | | | | |  |
|  | 1-6 days | | |  | 1-2 weeks | | | | |  | More than 2 weeks | | | | | |  | | |  |
| 18. | During the past month, has wrist/hand pain at any time made it difficult or impossible to do any of the following activities? | | | | | | | | | | | | | | | | | | |  |
|  | | | | | | | |  | | | | *No* | | | | *Difficult* | | *Impossible* | |  |
| a) Writing | | | | | | | | | | | |  | | | |  | |  | |  |
| b) Locking and unlocking doors | | | | | | | | | | | |  | | | |  | |  | |  |
| c) Opening bottles, jars or taps | | | | | | | | | | | |  | | | |  | |  | |  |
| d) Getting dressed | | | | | | | | | | | |  | | | |  | |  | |  |
| e) Doing the jobs that you normally do around the house | | | | | | | | | | | |  | | | |  | |  | |  |
| 19. | During the past month on how many days has wrist/hand pain prevented you from going to work? | | | | | | | | | | | | | | | | | | |  |
|  | 0 days |  | 1-5 days | | |  | | | More than 5 days | | | |  | | Not applicable because unemployed | | | |  |  |

**KNEE PAIN IN THE PAST MONTH**

| 20a) | Have you had pain in the knee in the area shown below which lasted for more than a day at any time during the past month? | | | | | | | | | | | | | | | | | |  |
| --- | --- | --- | --- | --- | --- | --- | --- | --- | --- | --- | --- | --- | --- | --- | --- | --- | --- | --- | --- |
| 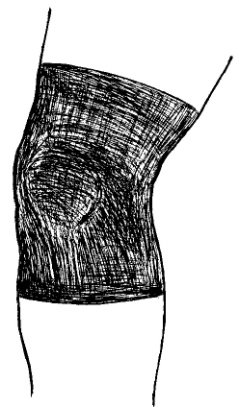 | | | | | | | | No | | | | | | Right knee only | | | | | |
|  |  |  |  |  |  |  |  | Left knee only | | | | | | Both knees | | | | | |
|  | | | | | | | | If ***NO,*** please go to question 23. If ***YES*** please continue. | | | | | | | | | | | |
| b) | If you add together all the days on which you have had knee pain, during the past month, how long a period would that make? | | | | | | | | | | | | | | | | | |  |
|  | 1-6 days | | |  | 1-2 weeks | | | |  | More than 2 weeks | | | | | |  | | |  |
| 21. | During the past month, has knee pain at any time made it difficult or impossible to do any of the following activities? | | | | | | | | | | | | | | | | | |  |
|  | | | | | | | |  | | | *No* | | | | *Difficult* | | *Impossible* | |  |
| a) Walking up and down stairs | | | | | | | | | | |  | | | |  | |  | |  |
| b) Walking on level ground | | | | | | | | | | |  | | | |  | |  | |  |
| c) Getting dressed | | | | | | | | | | |  | | | |  | |  | |  |
| d) Doing the jobs that you normally do around the house | | | | | | | | | | |  | | | |  | |  | |  |
| 22. | During the past month on how many days has knee pain prevented you from going to work? | | | | | | | | | | | | | | | | | |  |
|  | 0 days |  | 1-5 days | | |  | | More than 5 days | | | |  | | Not applicable because unemployed | | | |  |  |

| **SECTION SIX: YOUR HEALTH MORE GENERALLY** |
| --- |

**PAST 7 DAYS**

| 23. | Below is a list of problems people sometimes have. Please read each one carefully and circle the number that best describes HOW MUCH THAT PROBLEM HAS DISTRESSED OR BOTHERED YOU DURING THE **PAST 7 DAYS INCLUDING TODAY**  *Circle only* ***one number*** *for each problem and do not skip any items* | | | | | | | | | | | | | | | | | | | | |
| --- | --- | --- | --- | --- | --- | --- | --- | --- | --- | --- | --- | --- | --- | --- | --- | --- | --- | --- | --- | --- | --- |
|  | |  | *Not at all* | | |  | *A little bit* | | |  | *Moderately* | | |  | *Quite a bit* | | |  | *Extremely* | | |
| a) Faintness or dizziness | |  |  | 0 |  |  |  | 1 |  |  |  | 2 |  |  |  | 3 |  |  |  | 4 |  |
| b) Pains in the heart or chest | |  |  | 0 |  |  |  | 1 |  |  |  | 2 |  |  |  | 3 |  |  |  | 4 |  |
| c) Nausea or upset stomach | |  |  | 0 |  |  |  | 1 |  |  |  | 2 |  |  |  | 3 |  |  |  | 4 |  |
| d) Trouble getting your breath | |  |  | 0 |  |  |  | 1 |  |  |  | 2 |  |  |  | 3 |  |  |  | 4 |  |
| e) Numbness or tingling in parts of your body | |  |  | 0 |  |  |  | 1 |  |  |  | 2 |  |  |  | 3 |  |  |  | 4 |  |
| f) Feeling weak in parts of your body | |  |  | 0 |  |  |  | 1 |  |  |  | 2 |  |  |  | 3 |  |  |  | 4 |  |
| g) Hot or cold spells | |  |  | 0 |  |  |  | 1 |  |  |  | 2 |  |  |  | 3 |  |  |  | 4 |  |

**PAST MONTH**

| 24. | These questions are about how you feel and how things have been with you **during the past month.** For each question, please give the one answer that best describes how things have been for you during the past month. How much of the time during the **past month**:  *Circle* ***one number*** *on each line* | | | | | | |  |
| --- | --- | --- | --- | --- | --- | --- | --- | --- |
|  | | ***All of the time*** | ***Most of the time*** | ***A good bit of the time*** | ***Some of the time*** | ***A little of the time*** | ***None of the time*** | |
| a) Were you a happy person? | | 1 | 2 | 3 | 4 | 5 | 6 | |
| b) Have you felt calm and peaceful? | | 1 | 2 | 3 | 4 | 5 | 6 | |
| c) Have you been a very nervous person | | 1 | 2 | 3 | 4 | 5 | 6 | |
| d) Have you felt downhearted and low? | | 1 | 2 | 3 | 4 | 5 | 6 | |
| e) Have you felt so down that nothing could cheer you up? | | 1 | 2 | 3 | 4 | 5 | 6 | |

**PAST 12 MONTHS**

| 25. | Over the past 12 months, on how many days in total have you been prevented from going to work because of: | | | | | | | | |
| --- | --- | --- | --- | --- | --- | --- | --- | --- | --- |
| a) a problem with your back, neck, shoulder, elbow, wrist, hand or knees | | | | | | | | | |
|  | | 0 days |  | 1-5 days |  | 6-30 days |  | More than 30 days |  |
| b) other illness | | | | | | | | | |
|  | | 0 days |  | 1-5 days |  | 6-30 days |  | More than 30 days |  |
